# Investigating whether routinely collected biomarkers improve the prediction of hospital-acquired pressure injury occurrence: A retrospective cohort study

**DOI:** 10.1016/j.ijnsa.2025.100445

**Published:** 2025-11-01

**Authors:** Clair Merriman, Kathryn Suzann Taylor, Ria Betteridge, Neesha Oozageer Gunowa, Helen Walthall, Zoe Maunsell, Debra Jackson

**Affiliations:** a Oxford University Hospital NHS Foundation Trust, Oxford, UK; b Oxford Institute of Applied Health Research, Oxford Brookes University, Oxford, UK; c Nuffield Department of Primary Care Health Sciences, Oxford University, Oxford, UK; d The University of Surrey, Guildford, UK; e NIHR Oxford Biomedical Research Centre, UK; f The University of Sydney Australia, Australia

**Keywords:** Biomarkers, Braden Scale, Pressure ulcer, Risk Assessment

## Abstract

**Background:**

Despite being largely preventable, hospital-acquired pressure injuries remain a significant challenge in healthcare, contributing to prolonged hospital stays, increased patient morbidity, and substantial healthcare costs. Commonly used risk assessment tools have limited predictive accuracy, and early detection of hospital acquired pressure injuries often depends on subjective visual skin assessments. Emerging evidence suggests routinely collected biomarkers may offer an objective and reliable approach to predicting hospital acquired pressure injuries risk.

**Objective:**

To explore how biomarkers improve hospital acquired pressure injuries prediction.

**Design:**

Retrospective cohort study.

**Setting:**

Acute NHS Trust in England, UK.

**Participants:**

10,504 adult patients admitted to acute medical wards for at least 24 h in 2024.

**Methods:**

We considered the first hospital acquired pressure injuries and first record of other variables per hospital episode, measured at or soon after admission. Population characteristics of those who developed a hospital acquired pressure injuries or not were compared, overall and stratified by categories of length of stay (<6 days, ≥6 days), Braden, Malnutrition Universal Screening Tool and Mobility scores. Using multivariable logistic regression, we assessed the predictive value of the risk scores, adjusted for age and gender, and adding single biomarkers. Predictive performance was evaluated by discrimination and calibration. Analyses were exploratory. We used Stata v16 and R v4.4.

**Results:**

Median hospital stay for patients with hospital acquired pressure injuries (*n* = 293) was 18 days (interquartile range 12–31) compared with 5 days (2–11) for those without. Patients with hospital acquired pressure injuries were older than those without (84 (77–89) vs 78 (66–86) years. Levels of urea, C-reactive protein, and prothrombin time were significantly higher and albumin, haemoglobin and red blood cell count were significantly lower in those who developed hospital acquired pressure injuries. The incidence of hospital acquired pressure injuries was higher in those with longer hospital stays and increased across the risk score categories. Adjusting for age and gender, a unit increase in the Braden score reduced the odds of developing a hospital acquired pressure injuries by 15 %. The discrimination was adequate (AUC 0.72), but calibration was poor. Several individual biomarkers enhanced discrimination, but with miscalibration. Albumin was an independent predictor of hospital acquired pressure injuries in all models. The model with mobility adjusted for age and gender had adequate discrimination (AUC 0.71) and was well calibrated. Compared to those fully mobile, there was a sevenfold increase in the odds of hospital acquired pressure injuries in the immobile, and eightfold increase in those non-weight-bearing.

**Conclusions:**

This study provides insight into the potential role of biomarkers in enhancing hospital acquired pressure injuries risk prediction. Further research should investigate how using objective biomarkers, combined with risk scores, improves the prediction of hospital acquired pressure injuries.

**What is already known**

• Hospital aquired pressure injuries continue to be a harm risk for patients.
• Commonly used risk assessment tools have limited predictive accuracy and disadvantage people with non-Caucasian skin tones.
• Routinely collected biomarkers may be useful to identify patients at risk of hospital acquired pressure injuries and promote equity in skin assessment.

Alt-text: Unlabelled box

**What this paper adds**

• Analysis of 10,504 adult patients provides new evidence on the role of routinely collected biomarkers in predicting hospital-acquired pressure injuries.
• Several laboratory parameters were independently associated with hospital acquired pressure injuries risk, underscoring the potential of biomarkers as objective indicators beyond traditional risk scores.
• This study demonstrates how reliance on admission risk scores alone may underestimate risk in some patients, while integrating biomarkers could enhance sensitivity and promote equity in predictions, particularly where visual skin assessment is limited.
• This study provides a foundation for developing combined clinical–biochemical models of risk stratification that can support earlier, more tailored prevention strategies in acute care.

Alt-text: Unlabelled box

## 1 Background

Hospital-acquired pressure injuries continue to pose a significant challenge for patients and clinical teams (Kandula, 2025). hospital acquired pressure injuries have serious consequences for patients, including prolonged hospitalisation (Roussou et al., 2022), pain and discomfort (Jackson et al., 2017), increased susceptibility to infections, and complications such as sepsis or osteomyelitis, thereby exacerbating morbidity and mortality (Kandula 2025). Pressure injuries (PIs) remain among the most common and often preventable adverse events in hospital settings (Cordina et al. 2025;(Slawomirski, 2017). The global prevalence of hospital acquired pressure injuries among hospitalised adults is estimated to be approximately 12 % (Rodgers, Sim et al., 2021). In the US, hospital acquired pressure injuries affect over 2.5 million individuals, resulting in 60 000 deaths per year, similarly impacting 700 000 individuals in the United Kingdom (UK), resulting in 29 000 deaths (Padula and Pronovost 2018). Furthermore, hospital acquired pressure injuries raise concerns regarding patient care standards, with research findings suggesting a poor uptake of preventative measures (Fulbrook, Lovegrove et al., 2024).

hospital acquired pressure injuries also have cost implications. For example, the cost to the US health system was reported to be 25 billion USD annually in 2023–2024 (Simpson, 2023). Proportional costs are higher in the UK, where PIs were estimated to typically cost up to 4 % of the annual healthcare budget (Phillips, Humphreys et al., 2016). When community healthcare costs are added to hospital costs, PI treatment consumed up to £2.1 billion of the National Health Service (NHS) budget, most of which is associated with nursing costs (Bennett, Fromer et al., 2014).

Preventing hospital acquired pressure injuries is deemed a key role for nurses (Cordina, Rolls et al., 2025), with hospital acquired pressure injuries seen as an indicator of the quality of nursing care (Moya-Suárez, Canca-Sánchez et al., 2018). International PI prevention strategies have traditionally centred around risk assessment tools, for example, use of the Braden and Waterlow scores, to identify patients at risk of developing PI (Rodgers, Sim et al., 2021). These risk assessment tools rely on subjective assessment and do not necessarily provide nurses with a reliable prediction of the individual’s risk, due to the lack of validity and accuracy in practice (Park, Lee et al., 2015). Current methods for early PI identification rely heavily on visual skin inspection, which is less effective for individuals with dark skin tones, leading to disparities in detection and treatment (Oozageer Gunowa et al., 2024). Furthermore, damage to the skin or underlying tissue may not be visible to the naked eye, even though pathophysiological changes may already be occurring (Hajhosseini, Longaker et al., 2020).

Many hospital acquired pressure injuries are preventable, with early identification of risk and rapid implementation of preventative strategies being critical for their prevention. The growing number of people in hospital with complex and multi-factorial health needs makes traditional PI prevention strategies increasingly difficult to effectively implement. Emerging evidence suggests routinely collected objective measures, such as biomarkers, offer a more objective and reliable approach to identifying patients at risk of hospital acquired pressure injuries (Wang, et al., 2022; Elsorady and Nouh, 2023; Chang, et al., 2024). Therefore, these routinely measures may offer a solution for earlier detection of PI and increase the reliability of risk assessment procedures.

A patient’s clinical biomarkers, in combination with effective risk assessment tools, have been shown to enable early identification of individuals at risk of PI before tissue damage shows on the skin (Hatanaka, et al., 2008; Wang, et al., 2022; Elsorady and Nouh, 2023; Evanti, et al., 2023; Jin, Back et al., 2023; Chang, Jen et al., 2024; Ma, et al., 2024). Biomarkers are defined as indicators of the normal biological processes, pathogenic processes or responses to exposure or intervention (FDA-NIH Biomarkers Working Group, 2016). Clinical biomarkers can be categorised as diagnostic, monitoring, predictive, or susceptibility/risk-related (García-Gutiérrez, Navarrete et al., 2020). They may also be classified according to their underlying characteristics, including genetic, immune, metabolic, and nutritional domains (García-Gutiérrez, Navarrete et al., 2020).

It is not currently known which biomarkers are sensitive to early identification of PI. A systematic review and meta-analysis (Ma, et al., 2024) identified advanced age, male gender, hypoalbuminaemia, decreased haemoglobin concentration, diabetes, mechanical ventilation and length of hospital stay as risk factors for PIs. Similarly, a systematic review by Wang, et al. (2022) identified haemoglobin, CRP, and albumin levels, along with age and gender, as useful in identifying patients at risk of developing hospital acquired pressure injuries. Elsorady and Nouh (2023), identified similar findings, but also suggested co-morbidities, could also be valuable indicators. Others have reported blood results, combined with the patient's age, body mass index, mobility and length of stay are important predictors of hospital acquired pressure injuries (Jin, et al., 2023). Hatanaka, et al. (2008) study identified a combination of haemoglobin, CRP, albumin, age and gender as being more reliable than the Braden Score. Despite this current evidence base, researchers continue to recommend further research to confirm the predictive effect of biomarkers and to explore if risk assessment tools in conjunction with biomarkers improve predictive accuracy. In our study, we aimed to expand on previous research by exploring the use of routinely collected biomarkers to improve hospital acquired pressure injuries prediction and inform more effective hospital acquired pressure injuries prevention strategies.

## 2 Methods

### 2.1 Study design and participants

A retrospective descriptive cohort study design was used. The study took place in a large University Teaching NHS hospital Trust in the South of England. We included a consecutive sample of adult patients (18 years and over) who were admitted to 12 general medical acute inpatient clinical areas at the study site for at least 24 h between January 1st – December 31st, 2024. Patients were excluded if they had evidence of a PI on admission to the clinical area or within 24 h.

### 2.2 Data collection

Data extraction was completed by the Trust’s informatics team. The following biomarker data were extracted from the electronic patient record system for each patient: serum sodium, potassium, chloride, urea, creatinine, estimated glomerular filtration rate (eGFR, calculated using the CKD-EPI equation), albumin, alkaline phosphatase (ALP), amylase, C-reactive protein (CRP), haemoglobin, haematocrit, mean cell haemoglobin level (MCH), mean cell haemoglobin concentration (MCHC), mean platelet count, red blood cell count, and white cell count. International normalisation ratio (INR), prothrombin time, activated partial thromboplastin time (APTT), and Braden, Malnutrition Universal Screening Tool (MUST) and Mobility scores were also extracted. Scores and biomarkers were collected at or soon after hospital admission and then at each episode during admission until discharge. Collected at admission were the patients’ age, gender, ethnicity, body mass index (BMI), weight and height (to calculate missing BMI or replace implausible values), admission date, discharge date and name of clinical ward where the patient was admitted. For patients who developed a hospital acquired pressure injuries, the date of occurrence and the severity of tissue damage were documented, with injuries classified as Stage 2 or above and verified by a tissue viability nurse.

### 2.3 Operational definition of pressure injury

In this study, we defined PI in accordance with the International Pressure Ulcer/Injury Classification System developed by the National PI Advisory Panel (NPIAP), European Pressure Ulcer Advisory Panel (EPUAP), and Pan Pacific Pressure Injury Alliance (PPPIA) (NPIAP, 2025). A PI is “localized damage to the skin and/or underlying tissue, usually over a bony prominence, as a result of pressure, or pressure in combination with shear.”

### 2.4 Classification of pressure injury stages

All PIs were staged by tissue viability nurses using the NPIAP–EPUAP–PPPIA system. This classification includes:

• ***Stage 1:*** *Non-blanchable erythema of intact skin*
• ***Stage 2:*** *Partial-thickness skin loss with exposed dermis*
• ***Stage 3:*** *Full-thickness skin loss*
• ***Stage 4:*** *Full-thickness skin and tissue loss*
• ***Unstageable:*** *Obscured full-thickness skin and tissue loss*
• ***Deep tissue pressure injury:*** *Persistent non-blanchable deep red, maroon, or purple discolouration*

### 2.5 Data analysis

Descriptive statistics were derived by calculating the median and interquartile range for continuous variables and proportions for binary and categorical variables. These descriptive statistics summarised the characteristics of the study population, stratified by the presence or absence of a hospital acquired pressure injuries, overall and with further stratification by length of stay, Braden, MUST and mobility categories. Length of stay categories were ‘shorter’ (<6 days) and ‘longer’ (6 days or more). Braden score categories were 19+ (no risk), 15–18 (mild risk), 13–14 (moderate risk). 10–12 (high risk) and 9 or lower (severe risk). MUST scores were categorised into 0 (low risk), 1 (medium risk), and ≥2 (high risk). Mobility scores were categorised into 0 (fully mobile), 1 (mobility assistance) 2 (partially weight bearing); 3 (non-weight bearing, sit with support), 4 (Immobile/bedridden). Biomarkers with over 85 % missing data (amylase and potassium) were excluded. Groups with and without hospital acquired pressure injuries were compared by Mann Whitney U, Chi-squared test or a non-parametric Wilcoxon-type test for trend across ordered groups. An exploratory analysis of the predictors of hospital acquired pressure injuries was conducted using multivariable logistic regression, implemented by a generalised linear model. For this analysis, we excluded biomarkers with levels of missing data over 20 %, or if they were highly correlated with other biomarkers, with a correlation coefficient (CC) above 0.7. Variables with highly skewed distributions (CRP, ALP, CRP to albumin ratio and creatinine) were (natural) log-transformed before analysis. A base model was constructed to investigate the diagnostic value of the Braden score unadjusted, adjusted for age and gender, and with the following single biomarkers added as further predictors: albumin, log ALP, APTT, log creatinine, log CRP, log CRP to albumin (CRP[mg/L]/Albumin[g/L]) (Li et al., 2023), INR, haemoglobin, MCV, platelets, RBCC, sodium, WCC and MCHC. We also investigated the combination of predictors reported by Hatanaka, et al. (2008), namely age, gender, albumin, haemoglobin and log CRP. Two separate models included the MUST and Mobility categories, adjusted for age and gender. Goodness of fit was measured by the Hosmer and Lemeshow test (*p* < 0.05 indicates a poor fit) and predictive performance was evaluated by calibration plots and discrimination, by the area under the ROC curve (AUC, 0.5 represents no discrimination, 0.7–0.8 is adequate, 0.8–0.9 excellent, 1 is perfect discrimination) (Hosmer and Lemeshow, 2000). Descriptive analyses were based on available data without imputation, and prediction models were based on complete cases for each model. We only analysed the first hospital acquired pressure injuries and the first reading of other variables per hospital episode, with variables measured at or soon after hospital admission. The level of statistical significance was adjusted for multiple testing, using the Bonferroni correction to 0.05/21=0.002. All statistical analyses were conducted using Stata v16 (StataCorp, College Station, TX, USA) or R v4.4.

### 2.6 Ethical considerations

Approval for data extraction and analysis was obtained from the Trust Research and Development Department before data extraction began (approval number: 10,051). To protect patients’ identities, identifying information such as names was not extracted and NHS/MRN numbers were removed once data cleaning had been completed prior to data analysis. Given the study’s retrospective nature and classification as a service evaluation, informed consent was not required. All extracted data were password-protected, and documents were encrypted and managed throughout the data analysis process.

## 3 Results

**Fig. 1:**
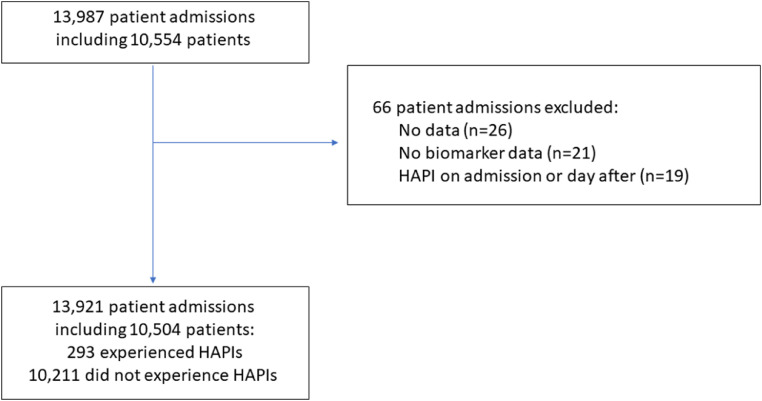
Flow chart.

We analysed 10,504 patients, including 293 patients who experienced at least one hospital acquired pressure injuries (Fig. 1). Of the biomarkers included in the descriptive analysis, levels of missing data were around 10 % for APTT, INR and prothrombin time and over 20 % for eGFR (Table 1). hospital acquired pressure injuries incidence stratified by injury severity is reported in Table 2. Most cases of hospital acquired pressure injuries (59 %) were classed as stage 2 injuries. Men and women had similar severity of hospital acquired pressure injuries and people who were noted to have non-white ethnicity (*n* = 10) had a higher proportion with severe hospital acquired pressure injuries than those with white ethnicity, although the small number limits interpretation. The severity of hospital acquired pressure injuries was higher in those classified with mild risk compared to those with moderate risk by the Braden score, and the severity was similar between those who used mobility aids (*n* = 119) and those who were immobile (*n* = 65).

**Table 1:** Missing data and ranges of patient characteristics.

Variable	N	Missing %	Minimum	Maximum
Age	10,504	0.0	18	104
Gender	10,504	0.0		
BMI	8535	18.7	9.2	160.0
Ethnicity	8775	16.5		
Albumin	10,256	2.4	10.0	57.0
ALP	10,242	2.5	19.0	3868.0
APTT	9422	10.3	14.2	166.1
Creatinine	10,376	1.2	13.0	1982.0
CRP	10,178	3.1	0.2	665.4
eGFR	8390	20.1	1.0	90.0
Haematocrit	10,384	1.1	0.1	0.6
Haemoglobin	10,381	1.2	27.0	214.0
INR	9485	9.7	0.9	17.0
MCV	10,384	1.1	50.5	156.5
MCHC	10,384	1.1	227.0	391.0
MCH	10,384	1.1	13.7	53.8
MPV	10,296	2.0	7.6	14.9
Platelets	10,377	1.2	1.0	1466.0
Prothrombin time	9468	9.9	9.1	117.0
RBCC	10,384	1.1	0.9	8.1
Sodium	10,377	1.2	100.0	186.0
Urea	10,372	1.3	1.1	92.9
WCC	10,384	1.1	0.0	394.9
Braden	9586	8.7	6	23
Mobility	8444	19.6	0	4
MUST	7944	24.4	0	4

Based on data for 10,504 patients. ALP, alkaline phosphatase; BMI, body mass index; APTT, activated partial thromboplastin time; CRP, C-reactive protein; eGFR, estimated glomerular filtration rate; MCH, mean cell haemoglobin; MCHC, mean cell haemoglobin concentration INR, International normalised ratio; MCV, mean cell volume; MPV, mean platelet volume; RBCC, red blood cell count; WCC, white cell count; MUST, Malnutrition Universal Screening Tool. Mobility codes: 0 fully mobile, 1 mobility assistance (frame/walking aid), 2 partially weight bearing; 3 non-weight bearing, sit with support, 4 Immobile/bedridden.

Normal ranges: APTT 20–30; Albumin 32–50; ALP 30–130;; CRP 0–5; Creatinine 49–90 (females), 64–104 (males); Haematocrit 0.36–0.46 (females), 0.40–0.50 (males); Haemoglobin 120–165 (females), 130–170 (males); INR 0.8–1.2; MCV 83–101; MPV 9–13; Platelets 150–400; Prothrombin time 9–12; RBCC 3.8–5.0 (females), 4.5–6.2 (males); Sodium 135–145; Urea 2.5–7.8 WCC 3.7–11.0; eGFR >60; MCHC 315–360; MCH 27.0–32.0;.

**Table 2:** Categories of hospital-acquired pressure injuries.

Variable	Stage 1	Stage 2	Stage 3	Stage 4	p-value
All	98 (33.5)	172 (58.7)	22 (7.5)	1 (0.3)	
Gender					
Female	60 (35.1)	97 (56.7)	14 (8.2)	0 (0.0)	
Male	38 (31.2)	75 (61.5)	8 (6.6)	1 (0.8)	0.58
Ethnicity					
White British	87 (36.3)	133 (55.4)	19 (7.9)	1 (0.4)	
Not White British	1 (10)	7 (70.0)	2 (20.0)	0 (0.0)	0.06
Missing	10 (23.3)	33 (74.4)	1 (2.3)	0 (0.0)	
Braden score					
No risk	14 (36.8)	23 (60.5)	1 (2.6)	0 (0.0)	
Mild risk	37 (30.6)	73 (60.3)	11 (9.1)	0 (0.0)	
Moderate risk	20 (35.7)	33 (58.9)	2 (3.6)	1 (1.8)	
High risk	24 (40.0)	31 (51.7)	5 (8.3)	0 (0.0)	
Severe risk	3 (16.7)	12 (66.7)	3 (16.7)	0 (0.0)	0.64
MUST score					
Low risk	73 (37.0)	109 (55.3)	14 (7.1)	1 (0.5)	
Medium risk	8 (19.5)	30 (73.2)	3 (7.3)	0 (0.0)	
High risk	15 (34.9)	23 (53.5)	5 (11.6)	0 (0.0)	0.22
Missing	2 (16.7)	10 (83.3)	0 (0.0)	0 (0.0)	
Mobility score					
Fully mobile	9 (50.0)	9 (50.0)	0 (0.0)	0.0(0)	
Uses mobility aids	36 (30.3)	76 (63.9)	7 (5.9)	0.0(0)	
Partially weight bearing	10 (33.3)	17 (56.7)	3 (10.0)	0.0(0)	
Non-weight bearing	17 (39.5)	19 (44.2)	6 (14.0)	1 (2.3)	
Immobile	19 (29.2)	42 (64.6)	4 (6.2)	0.0(0)	0.31
Missing	7 (38.9)	9 (50.0)	2 (11.1)	0.0(0)	

Based on the first injury in the admission for the 295 people who experienced hospital-acquired pressure injuries. Row percentages. Fisher’s exact tests were applied, ignoring the missing category.

**Fig. 2:**
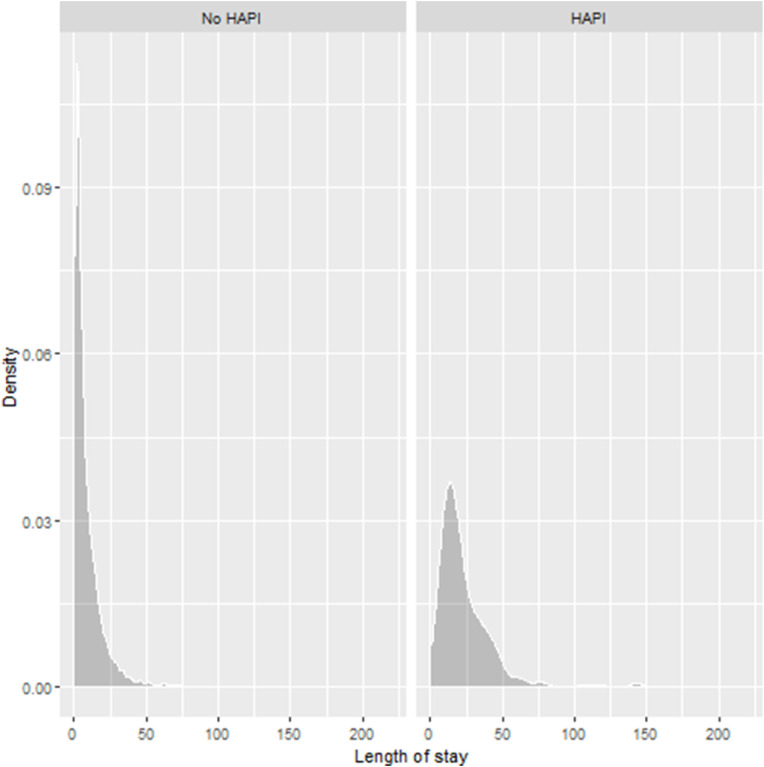
Density plots of length of hospital stay for those with and without hospital-acquired pressure injuries.

Table 3 presents the characteristics of patients, overall and stratified by hospital acquired pressure injuries incidence. Those with hospital acquired pressure injuries were significantly older than those without (hospital acquired pressure injuries: median (IQR) 84 (77–89); no hospital acquired pressure injuries 78(66–86) years). There were significant differences in the BMI profile, with a higher proportion of individuals with hospital acquired pressure injuries being underweight (18 % vs 6 %) and a lower proportion being obese (16 % vs 21 %). The biomarker distributions of those with hospital acquired pressure injuries differed from those without hospital acquired pressure injuries, with levels of albumin, haemoglobin and RBCC significantly lower and CRP, CRP to albumin, prothrombin time and urea significantly higher in those with hospital acquired pressure injuries and more variable. Braden scores at admission were significantly higher in those with hospital acquired pressure injuries (hospital acquired pressure injuries: median (IQR) 15 (12–17); no hospital acquired pressure injuries: 18 (15–20)), with a lower proportion with hospital acquired pressure injuries classed with no risk (13 % vs 38 %) and a higher proportion classed with moderate to severe risk (46 % vs 21 %). There were also significant differences in the MUST and Mobility scores of the two groups. Of those with hospital acquired pressure injuries, a higher proportion was classed by MUST scores at high risk (15 % vs 5 %), a higher proportion were immobile (22 % vs 10 %), and a lower proportion were fully mobile (6 % vs 27 %), compared to those without hospital acquired pressure injuries. The hospital stays of those with hospital acquired pressure injuries were significantly longer than those without hospital acquired pressure injuries (median IQR 5 (2–11) and 18 (11.75–31) respectively; Fig. 2).

**Table 3:** Characteristics of patients, overall and stratified by hospital-acquired pressure injury incidence.

	Overall	No hospital acquired pressure injuries	With hospital acquired pressure injuries	p
N	10,504	10,211	293	
Age	78.00 [65.00,86.00]	78.00 [65.00,86.00]	84.00 [77.00,89.00]	<0.001*
Age group				<0.001*
< 41 years	699 (6.7)	699 (6.8)	0 (0.0)	
41 to 60 years	1298 (12.4)	1286 (12.6)	12 (4.1)	
61 to 80 years	3991 (38.0)	3894 (38.1)	97 (33.1)	
>80 years	4516 (43.0)	4332 (42.4)	184 (62.8)	
Gender				0.011
Female	5345 (50.9)	5174 (50.7)	171 (58.4)	
Male	5159 (49.1)	5037 (49.3)	122 (41.6)	
Ethnic group				0.004
White British	7894 (75.2)	7654 (75.0)	240 (81.9)	
Not White British	881 (8.4)	871 (8.5)	10 (3.4)	
Missing	1729 (16.5)	1686 (16.5)	43 (14.7)	
BMI category				<0.001*
Underweight	636 (6.1)	581 (5.7)	55 (18.8)	
Healthy weight	3310 (31.5)	3193 (31.3)	117 (39.9)	
Overweight	2434 (23.2)	2375 (23.3)	59 (20.1)	
Obese	2167 (20.6)	2121 (20.8)	46 (15.7)	
Missing	1957 (18.6)	1941 (19.0)	16 (5.5)	
APTT [secs]	25.50 [23.30,28.48]	25.50 [23.20,28.40]	26.45 [23.63,29.90]	0.004
Albumin [g/L]	34.00 [30.00,38.00]	35.00 [30.00,38.00]	32.00 [27.00,35.00]	<0.001*
ALP [IU/L]	101.00 [79.00,135.00]	101.00 [79.00,135.00]	107.00 [83.00,150.00]	0.027
CRP [mg/L]	32.30 [6.70,107.40]	31.90 [6.60,106.70]	46.15 [13.25,122.22]	0.001*
CRP to Albumin [mg/g]	0.97 [0.18, 3.60]	0.95 [0.18, 3.57]	1.44 [0.40, 4.29]	<0.001*
Creatinine [µmol/L]	82.00 [63.00,115.00]	82.00 [63.00,114.00]	86.50 [63.75,135.00]	0.079
Haematocrit [L/L]	0.38 [0.34,0.42]	0.38 [0.34,0.42]	0.37 [0.33,0.41]	0.003
Haemoglobin [g/L]	127.00 [113.00,141.00]	127.00 [113.00,141.00]	123.00 [106.00,135.00]	<0.001*
INR [-]	1.10 [1.00,1.10]	1.10 [1.00,1.10]	1.10 [1.00,1.20]	<0.001*
MCV [fL]	91.80 [87.80,96.20]	91.80 [87.70,96.20]	92.60 [88.40,98.00]	0.004
MPV [fL]	10.10 [9.50,10.90]	10.10 [9.50,10.90]	10.20 [9.50,10.95]	0.746
Platelets [x10^9^/L]	246.00 [190.00,316.00]	246.50 [191.00,316.00]	235.00 [179.00,317.25]	0.491
Prothrombin time [secs]	11.10 [10.50,11.90]	11.10 [10.50,11.90]	11.40 [10.70,12.40]	<0.001*
RBCC [x10^12^/L]	4.21 [3.71,4.67]	4.21 [3.72,4.67]	4.05 [3.50,4.54]	<0.001*
Sodium [mmol/L]	137.00 [134.00,140.00]	137.00 [134.00,140.00]	136.00 [132.00,140.00]	0.073
Urea [mmol/L]	7.50 [5.40,11.30]	7.50 [5.30,11.20]	9.30 [6.80,14.80]	<0.001*
WCC [x10^9^/L]	10.20 [7.63,13.90]	10.20 [7.62,13.89]	10.16 [7.87,14.62]	0.570
eGFR [mL/min/1.73m^2^]	61.00 [40.00,79.00]	61.00 [40.00,79.00]	54.00 [34.00,76.00]	0.007
MCHC [g/L]	330.00 [320.00,339.00]	330.00 [320.00,339.00]	328.00 [318.00,337.00]	0.005
MCH [pg]	30.40 [28.90,31.90]	30.40 [28.90,31.90]	30.40 [29.00,32.00]	0.351
Braden score				<0.001*
No risk	3902 (37.1)	3864 (37.8)	38 (13.0)	
Low risk	3368 (32.1)	3247 (31.8)	121 (41.3)	
Moderate risk	1222 (11.6)	1166 (11.4)	56 (19.1)	
High risk	887 (8.4)	827 (8.1)	60 (20.5)	
Severe risk	217 (2.1)	199 (1.9)	18 (6.1)	
Missing	908 (8.6)	908 (8.9)	0 (0.0)	
MUST score				<0.001*
Low risk	6650 (63.3)	6453 (63.2)	197 (67.2)	
Medium risk	724 (6.9)	683 (6.7)	41 (14.0)	
High risk	581 (5.5)	538 (5.3)	43 (14.7)	
Missing	2549 (24.3)	2537 (24.8)	12 (4.1)	
Mobility score				<0.001*
Fully mobile	2795 (26.6)	2777 (27.2)	18 (6.1)	
Uses mobility aids	3304 (31.5)	3185 (31.2)	119 (40.6)	
Partial weight bearing	734 (7.0)	704 (6.9)	30 (10.2)	
Non-weight bearing	573 (5.5)	530 (5.2)	43 (14.7)	
Immobile	1057 (10.1)	992 (9.7)	65 (22.2)	
Missing	2041 (19.4)	2023 (19.8)	18 (6.1)	
Length of stay	5.00 [2.00,12.00]	5.00 [2.00,11.00]	18.00 [12.00,31.00]	<0.001*

Based on data for 10,504 patients. Row percentages. Reporting n ( %) or median (IQR). * Indicating *p* ≤ 0.002. ALP, Alkaline phosphatase; BMI, body mass index; APTT, activated partial thromboplastin time; CRP, C-reactive protein; eGFR, estimated glomerular filtration rate; MCH, mean cell haemoglobin; MCHC, mean cell haemoglobin concentration INR, International normalised ratio; MCV, mean cell volume; MPV, mean platelet volume; RBCC, red blood cell count; WCC, white cell count; MUST, Malnutrition Universal Screening Tool.

Normal ranges: APTT 20–30; Albumin 32–50; ALP 30–130; CRP 0–5; Creatinine 49–90 (females), 64–104 (males); Haematocrit 0.36–0.46 (females), 0.40–0.50 (males); Haemoglobin 120–165 (females), 130–170 (males); INR 0.8–1.2; MCV 83–101; MPV 9–13; Platelets 150–400; Prothrombin 9–12; RBCC 3.8–5.0 (females), 4.5–6.2 (males); Sodium 135–145; Urea 2.5–7.8 WCC 3.7–11.0; eGFR >60; MCHC 315–360; MCH 27.0–32.0;.

Supplementary Table 1 presents the patient characteristics stratified by time in hospital and hospital acquired pressure injuries incidence. The incidence of hospital acquired pressure injuries was 0.3 % for those with shorter hospital stays and 6 % for those with longer stays. The proportions who had hospital acquired pressure injuries increased across the MUST categories with 3.0 %, 5.7 % and 6.9 % for those classed as low, medium and high risk, respectively (Supplementary Table 3). Proportions with hospital acquired pressure injuries were 0.6 % for fully mobile patients, 3.6 % for those using mobility aids, 4.1 % for those with partial weight-bearing abilities, 7.5 % for those unable to bear weight and 6.1 % for those who were immobile (Supplementary Table 4).

Significantly higher proportions of patients with hospital acquired pressure injuries were in higher risk categories compared with those without hospital acquired pressure injuries. This pattern was consistent across stratifications by length of stay (Supplementary Table 1), including by Braden score (Supplementary Table 2), MUST score (Supplementary Table 3) and mobility categories (‘fully mobile’ and ‘uses mobility aids’) (Supplementary Table 4). There were significant differences in the length of hospital stay for patients stratified by each risk score (Supplementary Tables 2–4). Not all significant biomarker differences in the overall analysis remained consistent across the stratified analyses. Those that did apply included levels of albumin, which were significantly lower in those with hospital acquired pressure injuries with longer hospital stays (Supplementary Table 1), and in each of the lowest categories of the three risk scores (Supplementary Tables 2–4). Significantly higher levels of urea also applied to those stratified by length of stay (Supplementary Table 1) and for those in the low MUST category (Supplementary Table 3).

**Fig. 3:**
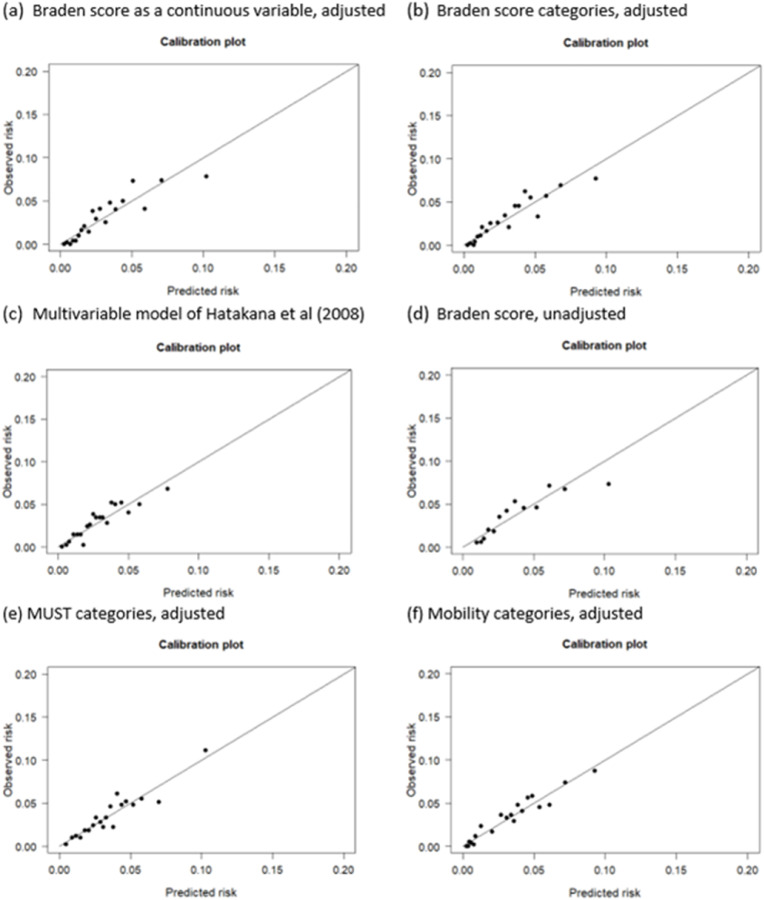
Calibration plots for selective models included in predictive modelling analysis.

Table 4 shows the results of the exploratory investigation into the predictive value of the risk scores with and without biomarkers. We excluded eGFR (>20 % missing), haematocrit (CC=0.97 with RBCC), prothrombin time (CC=0.92 with INR), urea (CC=0.74 with creatinine) and MCHC level (CC=0.86 with MCV). Adjusting for age and gender, for a unit increase in the Braden score, the odds of developing a hospital acquired pressure injuries reduced by a factor of 0.85 i.e. 15 % reduction in the odds. The discrimination was adequate with AUC 0.72 (0.70, 0.75), but the model fit was poor (Hosmer-Lemeshow *p* < 0.05). The calibration improved in a model with Braden score categories (Fig. 3a and b) and discrimination remained adequate (AUC 0.72 (0.69, 0.74)). In contrast with the findings of Hatanaka et al. (2008), the predictive value of the Braden score had a higher AUC compared to the model with predictors haemoglobin, log CRP, albumin, sex and gender (AUC 0.70 vs 0.68); however, the multivariable model had better calibration (Fig. 3c and d). Adding the Braden score as a further predictor produced a higher discrimination (0.73) but poor model fit (*p* < 0.05) and poor calibration. Albumin demonstrated independent predictive value in all multivariate analyses. Adding several biomarkers to the base model (AUC 0.724) improved the discrimination slightly, up to 0.733 by adding albumin to the model (Table 4).

**Table 4:** Results of multivariable logistic regression analysis for modelling the prediction of the development of hospital acquired pressure injuries.

Model	Odds ratio for risk score (95 % CI)	Odds ratio for biomarker	No with hospital acquired pressure injuries in analysis	Biomarker coefficient p value	Hosmer-Lemeshow p-value	Area under the curve
Braden score + Age + Gender	0.85 (0.83, 0.88)		293		0.01	0.724 (0.700, 0.748)
+ Albumin	0.87 (0.84, 0.90)	0.96 (0.95,0.98)	292	<0.001	0.01	0.733 (0.710, 0.757)
+ log ALP	0.85 (0.82, 0.88)	0.94 (0.75, 1.18)	292	0.62	0.1	0.724 (0.70 0, 0.748)
+ APTT	0.86 (0.83, 0.89)	1.01 (0.99, 1.02)	274	0.24	<0.001	0.727 (0.703, 0.752)
+ log(Creatinine)	0.85 (0.83, 0.88)	1.01 (0.81 1.25)	292	0.95	0.02	0.723 (0.699, 0.747)
+ log(CRP)	0.86 (0.83, 0.89)	1.05 (0.98, 1.14)	292	0.15	0.002	0.723 (0.698, 0.747)
+ log (CRP to Albumin)	0.86 (0.83, 0.89)	1.06 (0.99, 1.14)	291	0.09	0.008	0.723 (0.698, 0.747)
+ INR	0.86 (0.83, 0.89)	1.00 (0.79, 1.17)	273	0.98	0.003	0.726 (0.701, 0.751)
+ Haemoglobin	0.86 (0.83, 0.88)	1.00 (0.99, 1.00)	293	0.20	0.07	0.725 (0.702, 0.749)
+ MCV	0.85 (0.83, 0.88)	1.01 0.99, 1.03)	293	0.25	0.003	0.726 (0.702, 0.749)
+ Platelets	0.85 (0.83, 0.88)	1.00 (1.00, 100)	292	0.88	0.02	0.724 (0.699, 0.748)
+ RBCC	0.85 (0.83, 0.88)	0.87 (0.75, 1.02)	293	0.09	0.09	0.727 (0.703, 0.750)
+ Sodium	0.85 (0.82, 0.88)	0.98 (0.96, 1.00)	293	0.05	0.04	0.727 (0.704, 0.751)
+ WCC	0.85 (0.83, 0.88)	1.00 (0.99, 1.01)	293	0.77	0.02	0.724 (0.700, 0.748)
+ MCHC	0.85 (0.82, 0.88)	1.00 (0.99, 1.01)	293	0.64	0.06	0.725 (0.701, 0.749)
Braden score	0.84 (0.81, 0.86)		293		0.12	0.703 (0.677, 0.730)
Haemoglobin + log(CRP) + Albumin + Age + Gender (Hatanaka et al. 2008)		1.00 (1.00, 1.01) Hb 0.990 (0.91, 1.08) logCRP 0.94 (0.92, 0.96) Al	291	0.38 0.84 <0.001	0.19	0.682 (0.656, 0.709)
Braden + Haemoglobin + log(CRP) + Albumin + Age + Gender	0.87 (0.84, 0.90)	1.00 (1.00, 1.01) Hb 0.98 (0.90, 1.06) logCRP 0.96 (0.94, 0.99) Al	291	0.67 0.57 0.001	0.002	0.731 (0.708, 0.755)
Braden category + Age + Gender	2.83 (1.96, 4.18) Low vs No risk 3.47 (2.67, 5.37) Mod vs No risk 5.34 (3.50, 8.22) High vs No risk 7.19 (3.92, 12.75) Severe vs No risk		293		0.15	0.716 (0.691, 0.741)
MUST category + Age + Gender	1.63 (1.14, 2.30) Med vs Low 2.49 (1.74, 3.49) High vs Low		275		0.70	0.679 (0.649, 0.708)
Mobility + Age + Gender category	3.86 (2.36, 6.69) Uses aids vs Fully 4.47 (2.46, 8.36) Partial wt vs Fully 8.39 (4.79, 15.29) Non-wt vs Fully 6.81 (4.03, 12.09) Immobile vs Fully		291		0.62	0.712 (0.686, 0.737)

Hosmer and Lemeshow test p-value<0.05 indicates a poor fit. Area under the ROC curve 0.5 represents no discrimination, 0.7–0.8 adequate, 0.8–0.9 excellent, 1 perfect discrimination.

All dots lying along the diagonal would indicate perfect calibration, dots above the diagonal indicate overestimation of risks and dots below indicate underestimation of risks.

The odds of developing a hospital acquired pressure injuries for those classed as ‘Severe’ by the Braden score versus those classed as ‘No Risk’ increase by a factor of 6.84. The odds increase by a factor of 2.49 for those classed as high risk versus low risk by MUST scores. There was a sevenfold increase in the odds of those immobile, and the odds were over 8 times greater in the non-weight-bearing group compared to those fully mobile. The models including mobility and MUST scores had adequate discrimination and good model fit, and the model including mobility scores was well calibrated (Fig. 3e).

## 4 Discussion

This analysis of routinely collected data of around 10,500 patients provided new insights into the incidence and characteristics of hospital acquired pressure injuries, contributing to our understanding of risk factors. Study findings provide new evidence to highlight the multifactorial nature of hospital acquired pressure injuries risk, drawing attention to the potential for biomarker integration, the limitations of current risk assessment tools, and the interplay of individual factors, such as age, nutrition, mobility, and gender. These findings suggest hospital protocols and risk assessment models need refinement to better identify patients at heightened risk of hospital acquired pressure injuries and prevent pressure injuries in acute care settings.

Our findings support the findings of other researchers (Hatanaka, et al., 2008; Wang, et al., 2022; Elsorady and Nouh 2023; Evanti, et al., 2023; Jin, et al., 2023; Chang, et al., 2024; Ma, et al., 2024) who suggest that biomarkers can play a greater role in risk prediction models, providing clinicians with objective measures to guide early interventions. In our current study, distinct differences in biomarker profiles between patients who developed hospital acquired pressure injuries and those who did not are evident. Elevated levels of inflammatory and renal markers such as urea, CRP and prothrombin time were observed in patients with hospital acquired pressure injuries. Conversely, protective or functional indicators like albumin, haemoglobin, and red blood cell count were lower in the same group. Drawing on routinely collected biomarker data in hospitalised patients can not only provide valuable clinical insights into physiological risk factors for pressure injuries but also support more accurate and inclusive risk identification. This approach also has the potential to promote greater equity in care, particularly for patients with darker skin tones, who are disadvantaged by current assessment practices where the reliance is primarily on visual skin inspection alone (Oozageer Gunowa, et al., 2024).

The study confirms existing literature suggesting that older adults are at greater risk of developing hospital acquired pressure injuries (Fu et al., 2023), with the average age of affected patients in our study being higher (84 years median) than those who were unaffected (78 years). Additionally, a higher proportion of individuals with hospital acquired pressure injuries were underweight, while fewer were obese, suggesting that low body mass may be an important, and possibly underappreciated, risk factor. A longer length of hospital stay was also associated with hospital acquired pressure injuries. These findings support earlier findings of Fu et al. (2023), who also found PI was most likely to occur in inpatients with older age, longer hospital stay, and lower BMI. In our current study, the gender distribution also indicates a slight predominance of hospital acquired pressure injuries among women (58 % vs 51 %), albeit a non-significant difference, which could point to either physiological vulnerability or differences in care patterns. These findings differ from the review findings of Ma et al. (2024), who found males had a higher risk of PI, but these authors included studies from a broader clinical population than our current study.

We observed that the Braden risk score discriminated well between those with hospital acquired pressure injuries compared to those without, with the latter group having a higher median Braden score. However, our findings revealed the Braden score had poor calibration, overestimating risk for low observed risk groups and underestimating risk for medium observed risk. This was shown by patients classified as having a *mild risk* for hospital acquired pressure injuries by the Braden score experiencing more severe pressure injuries compared to those in the moderate risk category. This counterintuitive result raises important questions about both the reliability of the Braden score in accurately stratifying risk, particularly in distinguishing between mild and moderate-risk cases, and the potential for clinical complacency in lower-risk groups. Our findings concur with the concerns about the predictive validity of the Braden tool previously raised in the literature. A review that sought to examine the predictive and concurrent validity of the Braden scale in the long-term care environment raised concerns about its predictive accuracy and suggested the tool may overestimate risk, potentially leading to unnecessary interventions while failing to accurately identify those patients most in need of targeted preventative intervention (Wilchesky and Lungu, 2015). A study by Jin et al. (2015) assessed how well the Braden Scale identified patients at risk of developing pressure injuries by analysing longitudinal data from over 5900 hospital admissions. Their findings suggested that although the scale showed high sensitivity and negative predictive value, especially when using the lowest recorded score, a higher cut-off point than traditionally recommended may be more accurate for critically ill patients in real-world clinical settings.

It is possible that the Braden score, while widely used, lacks sufficient sensitivity for accurate prediction in certain populations, particularly older adults or those with subtle or fluctuating risk factors. For instance, patients may appear to have intact mobility or nutrition at the time of assessment but experience a rapid decline during hospitalisation. The score may also fail to account for compounding clinical factors contributing to skin breakdown such as inflammation or organ dysfunction, that are more accurately captured through biomarkers. Alternatively, this pattern may reflect a systemic issue in clinical response where patients labelled as *mild risk* are less likely to receive preventative measures such as regular repositioning, pressure-relieving equipment, or skin monitoring. In this scenario, risk classification could inadvertently lead to under-intervention, allowing seemingly low-risk patients to deteriorate without timely support.

These findings underscore the need to revisit how risk scores are interpreted and acted upon in clinical settings. Incorporating biomarker data into prediction models could improve risk stratification by adding objective physiological indicators, especially in cases where clinical presentation appears mild. Moreover, protocols may need to be adjusted to ensure even those deemed lower risk receive baseline preventative care, recognising hospital acquired pressure injuries development can occur across the risk spectrum. Classification by the Mobility score showed similar discriminatory ability to the Braden score but demonstrated better calibration. These findings suggest reliance on a single risk assessment tool may be insufficient and that combining tools or refining criteria may improve accuracy in clinical settings. Mobility limitations are well-known to contribute to PI risk, and this study supports that understanding, with higher proportions of those with hospital acquired pressure injuries with mobility problems compared to those without hospital acquired pressure injuries

The dataset revealed that among the small number of people where non-white ethnicity was noted (*n* = 10), hospital acquired pressure injuries tended to be more severe compared to those in Caucasian patients. While the sample size is limited, this finding aligns with existing literature suggesting that pressure injuries may go underdiagnosed or be detected later in individuals with dark skin tones due to the limitations of visual skin assessments. Subtle signs such as erythema or early tissue damage are often less visible on darker skin tones, increasing the risk of progression before intervention (Oozageer Gunowa, et al., 2024). This highlights the critical need for accurate recording of skin tone in medical records, and for more inclusive and diverse patient samples in future research to ensure findings are generalisable and risk models are equitable. It also reinforces the importance of developing robust, multimodal prediction tools that incorporate objective data, such as biomarkers, alongside clinical assessment. Reducing the overreliance on visual skin inspections alone could lead to earlier, more accurate detection of hospital acquired pressure injuries in all patients, regardless of skin tone.

Researchers have increasingly recognised the importance of combining clinical judgement with structured assessment tools when evaluating PI risk (Tao et al., 2024), as relying solely on one approach may not capture the complexity of patient needs. Previous studies have shown discrepancies in risk-level assessments and intervention decisions when nurses rely on structured tools versus clinical judgement alone (Fulbrook, et al. 2024), drawing attention to the need for a balanced approach combing clinical judgement, assessment evidence and other objective indicators (Tao et al., 2024). Our current study adds to this conversation, supporting the idea that routinely collected biomarkers, such as CRP, creatinine, and albumin, may provide valuable, objective data that could enhance the accuracy of PI risk stratification. By integrating biomarker data into the assessment process, clinicians may be better equipped to identify high-risk patients earlier, even when visual cues or assessment scores suggest otherwise. This reinforces the case for a more comprehensive approach to PI prevention, one that incorporates structured tools, professional judgement, and biomarker data to guide timely, tailored interventions. Nurses have a vital role in this approach, not only in conducting structured risk assessments but also in interpreting biomarkers to identify those at risk and implement early prevention strategies for hospital acquired pressure injuries. The integration of biomarkers into this application is a useful new approach for nurses to take.

### 4.1 Limitations and future research

There are some limitations to our study. Data were collected from a single NHS acute trust, with admissions to general medical wards only. Therefore, the diversity of the population may not be representative. Local care pathways, documentation practices, and prevention resources may differ from other hospitals and from surgical, ICU, or rehabilitation settings, limiting external validity. In addition, complete-case modelling excluded people with missing covariates, which could introduce selection bias if data were not missing at random. Complete-case analysis also excluded a few people who had experienced hospital acquired pressure injuries, and although this effect was minimised by including all available data for each model, it meant some comparisons of model performance were based on data with different numbers of people with hospital acquired pressure injuries. As this was a retrospective data set, there was missing data that could not be retrieved, which limited the scope of the analysis to preclude analysis by ethnicity and obesity. The data set did not include important confounding variables, including the cause of hospitalisation, chronic diseases and a comorbidity index. Biomarkers such as CRP, creatinine, and albumin are associated with both illness severity and hospital acquired pressure injuries risk and may therefore confound observed associations. We partially addressed this by adjusting for age and sex and by evaluating risk scores (Braden, MUST, mobility) to capture functional and nutritional status; however, we lacked standardised severity indices, so residual confounding is likely. Future analyses should incorporate validated severity measures and comorbidity burden, and where biomarkers are used as candidate predictors, consider models designed to distinguish their role as confounders from their role as predictors.

Multicentre replication, inclusion of additional specialities, and multiple imputation for missing data are also warranted to improve generalisability and reduce selection bias. Sample size limitations and the low prevalence of hospital acquired pressure injuries ruled out the consideration of a model containing all biomarkers (Sun et al., 1996; Steyerberg, 2009). Therefore, the conclusions of predictive value from this exploratory study would need to be verified by a more comprehensive study, where biomarkers not considered important in this study may, in combination with other predictors, be shown to the more relevant. There was a time-at-risk bias as patients who remain in hospital longer have more opportunity to develop a hospital acquired pressure injuries. Although we restricted predictors to measurements taken at (or soon after) admission and reported results stratified by length of stay, residual time-at-risk bias may remain. Because we used logistic regression to model the probability of a binary outcome (hospital acquired pressure injuries vs no hospital acquired pressure injuries), we could not fully account for differing exposure times. Future work could account for differing exposure times, censoring and competing risks with Cox regression models.

While our findings raised questions about the Braden scale’s predictive validity, we acknowledge our analysis has limitations in this regard. The number of hospital acquired pressure injuries cases in our dataset was relatively small, which reduces statistical power and limits the generalisability of conclusions about the performance of the risk tools. Moreover, the Braden scale, despite recognised disadvantages such as subjectivity and variability in practice, remains a sensitive and multidimensional tool that considers mobility, sensory perception, moisture, nutrition, and activity. Importantly, best practice recommends the Braden scale assessments be repeated at regular intervals throughout hospitalisation to reflect changes in patient status; however, in our study, only admission scores were available. This likely reduced the ability of the tool to reflect dynamic changes in risk during admission. In addition, our comparison was restricted to routinely collected laboratory indices and did not include other clinical variables such as comorbidities or severity of illness, which further limits interpretation. Therefore, while our findings suggest biomarkers may add value to risk prediction, they should not be viewed as a replacement for multidimensional assessment tools such as the Braden scale. Instead, future work should examine how biomarkers can complement repeated, structured clinical assessments to provide a more accurate and responsive estimate of hospital acquired pressure injuries risk.

## 5 Conclusions

This study provides useful insights into the potential role of biomarkers in enhancing hospital acquired pressure injuries risk prediction. We add to the current evidence base by demonstrating how routinely collected biomarkers provide valuable, objective data. Integrating these biomarkers into assessment process, may helpclinicians identify high-risk patients earlier, even when visual cues or assessment scores suggest otherwise. Further research is needed to fully understand the value of using biomarkers to identify patients at high risk of hospital acquired pressure injuries early, by a comprehensive investigation of the predictive performance of risk scores combined with biomarkers. Importantly, the incorporation of biomarkers could help overcome inequities in current practice, particularly the limitations of visual skin assessment in patients with darker skin tones. Ultimately, combining biomarkers with structured risk scores and clinical judgement could offer a pathway toward more accurate, equitable, and timely prevention of hospital acquired pressure injuries in acute care.

## Registration

Registration number, the registry, registration date, and date of first recruitment *must be provided for clinical trials. Not applicable*

## Social media abstract

can routinely collected biomarkers improve the prediction of hospital-acquired pressure injury occurrence: @ouh_research @ClairMerriman9 @debraejackson @debrae.bsky.social *@ne55hao*

## Funding

*This work was supported by the National Institute of Health and Care Research, Research Capacity Funding 2024/25 Focused call [grant number RCF24_F010].*

## CRediT authorship contribution statement

**Clair Merriman:** Writing – review & editing, Writing – original draft, Visualization, Validation, Project administration, Methodology, Investigation, Funding acquisition, Formal analysis, Data curation, Conceptualization. **Kathryn Suzann Taylor:** Writing – review & editing, Writing – original draft, Visualization, Validation, Methodology, Investigation, Formal analysis, Data curation, Conceptualization. **Ria Betteridge:** Writing – review & editing, Validation, Project administration, Methodology, Investigation, Data curation, Conceptualization. **Neesha Oozageer Gunowa:** Writing – review & editing, Methodology, Investigation, Conceptualization. **Helen Walthall:** Writing – review & editing, Visualization, Validation, Methodology, Investigation, Formal analysis, Data curation, Conceptualization. **Zoe Maunsell:** Writing – review & editing, Methodology, Formal analysis, Conceptualization. **Debra Jackson:** Writing – review & editing, Writing – original draft, Visualization, Validation, Methodology, Investigation, Data curation, Conceptualization.

## Declaration of competing interest

The authors declare the following financial interests/personal relationships which may be considered as potential competing interests:

Clair Merriman reports financial support was provided by National Institutes of Health. If there are other authors, they declare that they have no known competing financial interests or personal relationships that could have appeared to influence the work reported in this paper.

## Appendix Supplementary materials

Image, application 1
