# Supplementary material for: Investigating whether routinely collected biomarkers improve the prediction of hospital-acquired pressure injury occurrence: A retrospective cohort study
Source: Int J Nurs Stud Adv. 2025 Nov 1;9:100445. doi: 10.1016/j.ijnsa.2025.100445 (PMC12664353; doi:10.1016/j.ijnsa.2025.100445)
Supplement: Supplementary file 1 [file mmc1.docx]

Supplementary Table 1. Characteristics of patient admissions, stratified by length of stay category and hospital-acquired pressure injury incidence

|  | Length of stay < 6 days |  |  | Length of stay ≥ 6 days |  |  |
| --- | --- | --- | --- | --- | --- | --- |
|  | No HAPI | HAPI | p | No HAPI | HAPI | p |
| N* | 5462 | 16 |  | 4749 | 277 |  |
| Age | 76.00 [61.00,84.00] | 84.00 [81.50,91.25] | 0.001 | 80.00 [70.00,87.00] | 84.00 [77.00,89.00] | <0.001 |
| Age group |  |  | 0.015 |  |  | <0.001 |
| < 41 years | 568 (10.4) | 0 (0.0) |  | 131 (2.8) | 0 (0.0) |  |
| 41 to 60 years | 789 (14.4) | 1 (6.2) |  | 497 (10.5) | 11 (4.0) |  |
| 61 to 80 years | 2108 (38.6) | 3 (18.8) |  | 1786 (37.6) | 94 (33.9) |  |
| >80 years | 1997 (36.6) | 12 (75.0) |  | 2335 (49.2) | 172 (62.1) |  |
| Gender |  |  | 0.823 |  |  | 0.02 |
| Female | 2748 (50.3) | 9 (56.2) |  | 2426 (51.1) | 162 (58.5) |  |
| Male | 2714 (49.7) | 7 (43.8) |  | 2323 (48.9) | 115 (41.5) |  |
| Ethnic group |  |  | 0.553 |  |  | 0.01 |
| White British | 4067 (74.5) | 13 (81.2) |  | 3587 (75.5) | 227 (81.9) |  |
| Not White British | 523 (9.6) | 2 (12.5) |  | 348 (7.3) | 8 (2.9) |  |
| Missing | 872 (16.0) | 1 (6.2) |  | 814 (17.1) | 42 (15.2) |  |
| BMI category |  |  | 0.101 |  |  | <0.001 |
| Underweight | 197 (3.6) | 2 (12.5) |  | 384 (8.1) | 53 (19.1) |  |
| Healthy weight | 1357 (24.8) | 4 (25.0) |  | 1836 (38.7) | 113 (40.8) |  |
| Overweight | 1148 (21.0) | 6 (37.5) |  | 1227 (25.8) | 53 (19.1) |  |
| Obese | 1089 (19.9) | 1 (6.2) |  | 1032 (21.7) | 45 (16.2) |  |
| Missing | 1671 (30.6) | 3 (18.8) |  | 270 (5.7) | 13 (4.7) |  |
| APTT [secs] | 25.30 [23.00,27.90] | 24.50 [23.00,28.10] | 0.649 | 25.90 [23.40,29.10] | 26.50 [23.65,29.90] | 0.071 |
| Albumin [g/L] | 36.00 [31.00,40.00] | 33.50 [25.50,37.25] | 0.064 | 33.00 [28.00,37.00] | 31.50 [27.00,35.00] | <0.001* |
| ALP [IU/L] | 97.00 [77.00,128.00] | 94.50 [72.25,106.50] | 0.244 | 105.00 [82.00,142.00] | 108.00 [83.75,151.25] | 0.277 |
| CRP [mg/L] | 25.60 [4.90,92.50] | 56.60 [17.45,155.07] | 0.148 | 40.35 [9.50,122.30] | 45.30 [13.05,121.58] | 0.239 |
| CRP to Albumin [mg/g] | 0.75 [0.13,2.95] | 1.89 [0.46,5.38] | 0.125 | 1.23 [0.28,4.30] | 1.43 [0.39,4.27] | 0.159 |
| Creatinine [µmol/L] | 80.00 [63.00,108.00] | 112.00 [74.25,160.25] | 0.104 | 84.00 [63.00,121.00] | 85.00 [63.00,134.25] | 0.453 |
| Haematocrit [L/L] | 0.39 [0.35,0.43] | 0.41 [0.38,0.44] | 0.095 | 0.38 [0.34,0.42] | 0.37 [0.33,0.41] | 0.032 |
| Haemoglobin [g/L] | 129.00 [115.00,142.00] | 133.00 [123.50,142.75] | 0.341 | 125.00 [110.00,139.00] | 122.00 [106.00,135.00] | 0.014 |
| INR [-] | 1.00 [1.00,1.10] | 1.10 [1.00,1.10] | 0.493 | 1.10 [1.00,1.20] | 1.10 [1.00,1.20] | 0.057 |
| MCV [fL] | 91.30 [87.40,95.70] | 94.20 [89.62,96.62] | 0.319 | 92.30 [88.20,96.70] | 92.60 [88.40,98.00] | 0.107 |
| MPV [fL] | 10.10 [9.50,10.90] | 10.55 [9.70,11.48] | 0.229 | 10.20 [9.50,10.90] | 10.20 [9.45,10.90] | 0.951 |
| Platelets [x10^9^/L] | 246.00 [193.00,312.00] | 230.00 [216.25,321.75] | 0.625 | 247.00 [188.00,320.00] | 236.00 [175.75,312.50] | 0.44 |
| Prothrombin time [secs] | 11.00 [10.50,11.70] | 11.20 [10.70,11.90] | 0.377 | 11.20 [10.60,12.10] | 11.40 [10.70,12.40] | 0.043 |
| RBCC [x10^12^/L] | 4.29 [3.79,4.73] | 4.46 [4.25,5.22] | 0.098 | 4.11 [3.63,4.58] | 4.03 [3.44,4.50] | 0.013 |
| Sodium [mmol/L] | 137.00 [134.00,140.00] | 140.50 [135.25,144.25] | 0.047 | 137.00 [133.00,140.00] | 136.00 [132.00,139.00] | 0.095 |
| Urea [mmol/L] | 6.90 [5.00,10.20] | 12.80 [7.75,17.47] | <0.001* | 8.20 [5.80,12.10] | 9.10 [6.70,14.30] | <0.001* |
| WCC [x10^9^/L] | 10.03 [7.58,13.53] | 11.55 [8.89,19.42] | 0.059 | 10.41 [7.70,14.37] | 10.14 [7.83,14.39] | 0.681 |
| eGFR [mL/min/1.73m^2^] | 62.00 [43.00,79.00] | 47.00 [31.50,62.00] | 0.106 | 59.00 [38.00,79.00] | 55.00 [34.00,76.00] | 0.102 |
| MCHC [g/L] | 331.00 [321.00,340.00] | 328.00 [315.50,335.75] | 0.201 | 329.00 [319.00,338.00] | 328.00 [318.00,337.00] | 0.13 |
| MCH [pg] | 30.30 [28.90,31.80] | 30.40 [28.88,32.28] | 0.893 | 30.50 [28.90,32.00] | 30.40 [29.00,32.00] | 0.645 |
| Braden score |  |  | <0.001* |  |  | <0.001* |
| No risk | 2487 (45.5) | 1 (6.2) |  | 1377 (29.0) | 37 (13.4) |  |
| Low risk | 1367 (25.0) | 8 (50.0) |  | 1880 (39.6) | 113 (40.8) |  |
| Moderate risk | 352 (6.4) | 0 (0.0) |  | 814 (17.1) | 56 (20.2) |  |
| High risk | 277 (5.1) | 5 (31.2) |  | 550 (11.6) | 55 (19.9) |  |
| Severe risk | 90 (1.6) | 2 (12.5) |  | 109 (2.3) | 16 (5.8) |  |
| Missing | 889 (16.3) | 0 (0.0) |  | 19 (0.4) | 0 (0.0) |  |
| MUST score |  |  | 0.602 |  |  | <0.001* |
| Low risk | 2883 (52.8) | 10 (62.5) |  | 3570 (75.2) | 187 (67.5) |  |
| Medium risk | 231 (4.2) | 1 (6.2) |  | 452 (9.5) | 40 (14.4) |  |
| High risk | 167 (3.1) | 1 (6.2) |  | 371 (7.8) | 42 (15.2) |  |
| Missing | 2181 (39.9) | 4 (25.0) |  | 356 (7.5) | 8 (2.9) |  |
| Mobility score |  |  | <0.001* |  |  | <0.001* |
| Fully mobile | 1848 (33.8) | 1 (6.2) |  | 929 (19.6) | 17 (6.1) |  |
| Uses mobility aids | 1229 (22.5) | 5 (31.2) |  | 1956 (41.2) | 114 (41.2) |  |
| Partial weight bearing | 198 (3.6) | 3 (18.8) |  | 506 (10.7) | 27 (9.7) |  |
| Non-weight bearing | 136 (2.5) | 0 (0.0) |  | 394 (8.3) | 43 (15.5) |  |
| Immobile | 286 (5.2) | 5 (31.2) |  | 706 (14.9) | 60 (21.7) |  |
| Missing | 1765 (32.3) | 2 (12.5) |  | 258 (5.4) | 16 (5.8) |  |
| Length of stay | 2.00 [2.00,4.00] | 5.00 [4.00,5.00] | <0.001* | 12.00 [8.00,19.00] | 18.00 [13.00,31.00] | <0.001* |

Based on data for 10,504 patients. Reporting n(%) or median (IQR). Row percentages. * Indicating p≤0.002. Based on data for 10,504 patients. ALP, alkaline phosphatase; BMI, body mass index; APTT, activated partial thromboplastin time; CRP, C-reactive protein; eGFR, estimated glomerular filtration rate; MCH, mean cell haemoglobin; MCHC, mean cell haemoglobin concentration INR, International normalised ratio; MCV, mean cell volume; MPV, mean platelet volume; RBCC, red blood cell count; WCC, white cell count; MUST, Malnutrition Universal Screening Tool. Mobility codes: 0 fully mobile, 1 mobility assistance (frame/walking aid), 2 partially weight bearing; 3 non-weight bearing, sit with support, 4 Immobile/bedridden.

Normal ranges: APTT 20-30; Albumin 32-50; ALP 30-130;; CRP 0-5; Creatinine 49-90 (females), 64-104 (males); Haematocrit 0.36-0.46 (females), 0.40-0.50 (males); Haemoglobin 120-165 (females), 130-170 (males); INR 0.8-1.2; MCV 83-101; MPV 9-13; Platelets 150-400; Prothrombin time 9-12; RBCC 3.8-5.0 (females), 4.5-6.2 (males); Sodium 135-145; Urea 2.5-7.8 WCC 3.7-11.0; eGFR >60; MCHC 315-360; MCH 27.0-32.0;

Supplementary Table 2. Characteristics of patient admissions, stratified by Braden score category and hospital-acquired pressure injury incidence

|  | Low risk |  |  | Moderate risk |  |  | High risk |  |  | Severe risk |  |  |
| --- | --- | --- | --- | --- | --- | --- | --- | --- | --- | --- | --- | --- |
|  | No HAPI | HAPI | p | No HAPI | HAPI | p | No HAPI | HAPI | p | No HAPI | HAPI | p |
| N* | 3247 | 121 |  | 1166 | 56 |  | 827 | 60 |  | 199 | 18 |  |
| Age | 81.00 [72.00,88.00] | 85.00 [79.00,90.00] | <0.001* | 83.00 [76.00,89.00] | 84.00 [73.00,90.00] | 0.913 | 84.00 [75.00,88.50] | 84.50 [79.00,89.25] | 0.156 | 81.00 [72.00,87.00] | 78.00 [67.00,84.50] | 0.281 |
| Age group |  |  | <0.001* |  |  | 0.791 |  |  | 0.399 |  |  | 0.147 |
| < 41 years | 74 (2.3) | 0 (0.0) |  | 14 (1.2) | 0 (0.0) |  | 15 (1.8) | 0 (0.0) |  | 8 (4.0) | 0 (0.0) |  |
| 41 to 60 years | 259 (8.0) | 3 (2.5) |  | 61 (5.2) | 4 (7.1) |  | 44 (5.3) | 1 (1.7) |  | 15 (7.5) | 3 (16.7) |  |
| 61 to 80 years | 1209 (37.2) | 31 (25.6) |  | 397 (34.0) | 19 (33.9) |  | 264 (31.9) | 22 (36.7) |  | 76 (38.2) | 10 (55.6) |  |
| >80 years | 1705 (52.5) | 87 (71.9) |  | 694 (59.5) | 33 (58.9) |  | 504 (60.9) | 37 (61.7) |  | 100 (50.3) | 5 (27.8) |  |
| Gender |  |  | 0.028 |  |  | 0.670 |  |  | 0.014 |  |  | 0.636 |
| Female | 1668 (51.4) | 75 (62.0) |  | 607 (52.1) | 27 (48.2) |  | 394 (47.6) | 39 (65.0) |  | 95 (47.7) | 7 (38.9) |  |
| Male | 1579 (48.6) | 46 (38.0) |  | 559 (47.9) | 29 (51.8) |  | 433 (52.4) | 21 (35.0) |  | 104 (52.3) | 11 (38.9) |  |
| Ethnic group |  |  | 0.033 |  |  | 0.282 |  |  | 0.641 |  |  | 0.725 |
| White British | 2520 (77.6) | 106 (87.6) |  | 927 (79.5) | 47 (83.9) |  | 613 (74.1) | 43 (71.7) |  | 157 (78.9) | 13 (72.2) |  |
| Not White British | 221 (6.8) | 4 (3.3) |  | 50 (4.3) | 0 (0.0) |  | 57 (6.9) | 3 (5.0) |  | 13 (6.5) | 2 (11.1) |  |
| Missing | 506 (15.6) | 11 (9.1) |  | 189 (16.2) | 9 (16.1) |  | 157 (19.0) | 14 (23.3) |  | 29 (14.6) | 3 (16.7) |  |
| BMI category |  |  | <0.001* |  |  | 0.057 |  |  | 0.003 |  |  | 0.45 |
| Underweight | 229 (7.1) | 23 (19.0) |  | 82 (7.0) | 9 (16.1) |  | 76 (9.2) | 14 (23.3) |  | 19 (9.5) | 1 (5.6) |  |
| Healthy weight | 1138 (35.0) | 53 (43.8) |  | 441 (37.8) | 25 (44.6) |  | 303 (36.6) | 21 (35.0) |  | 68 (34.2) | 7 (38.9) |  |
| Overweight | 803 (24.7) | 21 (17.4) |  | 258 (22.1) | 8 (14.3) |  | 160 (19.3) | 14 (23.3) |  | 36 (18.1) | 5 (27.8) |  |
| Obese | 701 (21.6) | 22 (18.2) |  | 237 (20.3) | 8 (14.3) |  | 142 (17.2) | 6 (10.0) |  | 20 (10.1) | 3 (16.7) |  |
| Missing | 376 (11.6) | 2 (1.7) |  | 148 (12.7) | 6 (10.7) |  | 146 (17.7) | 5 (8.3) |  | 56 (28.1) | 2 (11.1) |  |
| APTT [secs] | 25.80 [23.40,28.90] | 26.40 [23.70,30.18] | 0.104 | 26.10 [23.70,29.20] | 25.75 [23.48,29.95] | 0.795 | 26.20 [23.60,30.30] | 26.85 [24.08,29.60] | 0.731 | 26.55 [23.10,31.33] | 26.05 [23.60,27.72] | 0.395 |
| Albumin [g/L] | 34.00 [29.00,37.00] | 31.00 [27.00,36.00] | 0.001* | 32.00 [28.00,36.00] | 32.00 [27.75,35.00] | 0.246 | 31.50 [27.00,36.00] | 31.00 [26.00,34.00] | 0.318 | 29.00 [25.00,35.00] | 30.00 [24.00,34.50] | 0.743 |
| ALP PT [IU/L] | 104.00 [80.00,139.00] | 109.00 [86.00,143.00] | 0.207 | 108.00 [86.00,145.00] | 101.50 [79.25,154.50] | 0.509 | 109.00 [83.00,145.75] | 114.00 [82.00,157.25] | 0.976 | 115.00 [85.00,176.00] | 104.50 [93.50,167.25] | 0.85 |
| CRP [mg/L] | 33.60 [7.30,108.10] | 35.80 [10.20,116.30] | 0.476 | 44.10 [14.00,119.10] | 55.70 [15.28,113.40] | 0.615 | 57.50 [14.53,138.85] | 45.10 [16.35,146.80] | 0.692 | 79.10 [26.35,176.80] | 140.65 [25.80,247.10] | 0.292 |
| CRP to Albumin [mg/g] | 1.05 [0.21,3.61] | 1.29 [0.34,3.90] | 0.358 | 1.39 [0.41,4.17] | 1.66 [0.50,3.78] | 0.615 | 1.91 [0.45,5.19] | 1.52 [0.50,4.35] | 0.709 | 2.77 [0.83,6.58] | 4.52 [0.74,8.40] | 0.303 |
| Creatinine [µmol/L] | 84.00 [63.00,120.00] | 85.00 [65.00,133.00] | 0.653 | 88.00 [64.00,129.00] | 80.50 [57.25,124.25] | 0.133 | 90.00 [66.00,135.00] | 110.00 [74.50,160.00] | 0.1 | 94.50 [63.00,133.00] | 93.00 [69.25,178.50] | 0.55 |
| Haematocrit [L/L] | 0.38 [0.34,0.42] | 0.37 [0.33,0.40] | 0.053 | 0.38 [0.34,0.42] | 0.38 [0.33,0.42] | 0.965 | 0.38 [0.34,0.42] | 0.36 [0.33,0.43] | 0.659 | 0.38 [0.33,0.42] | 0.38 [0.32,0.45] | 0.959 |
| Haemoglobin [g/L] | 125.00 [111.00,138.00] | 123.00 [106.00,131.00] | 0.035 | 124.00 [109.00,137.25] | 125.50 [105.50,137.25] | 0.914 | 124.00 [109.00,139.00] | 117.50 [105.50,137.25] | 0.407 | 120.50 [107.25,136.75] | 123.50 [102.75,137.75] | 0.966 |
| INR [-] | 1.10 [1.00,1.20] | 1.10 [1.00,1.10] | 0.537 | 1.10 [1.00,1.20] | 1.10 [1.00,1.30] | 0.141 | 1.10 [1.00,1.20] | 1.10 [1.00,1.20] | 0.981 | 1.10 [1.00,1.20] | 1.10 [1.00,1.10] | 0.098 |
| MCV [fL] | 92.20 [88.20,96.40] | 93.00 [89.40,98.00] | 0.014 | 92.45 [88.30,97.20] | 92.55 [88.38,97.93] | 0.702 | 93.60 [89.00,97.80] | 91.95 [87.40,95.50] | 0.058 | 93.25 [88.30,98.38] | 93.50 [86.85,98.12] | 0.883 |
| MPV [fL] | 10.20 [9.50,10.90] | 10.20 [9.50,10.93] | 0.854 | 10.20 [9.50,10.90] | 10.20 [9.45,10.80] | 0.776 | 10.20 [9.60,11.00] | 10.45 [9.57,11.12] | 0.583 | 10.20 [9.60,11.00] | 9.80 [9.07,10.95] | 0.166 |
| Platelets [x10^9^/L] | 244.00 [189.00,316.00] | 226.00 [163.00,298.00] | 0.106 | 243.00 [186.00,313.00] | 266.00 [191.50,333.50] | 0.125 | 248.00 [184.00,320.00] | 247.00 [190.50,322.00] | 0.88 | 258.00 [195.00,355.50] | 286.00 [187.75,397.75] | 0.629 |
| Prothrombin time [secs] | 11.10 [10.60,12.00] | 11.40 [10.50,11.95] | 0.515 | 11.30 [10.70,12.30] | 11.45 [10.88,13.20] | 0.12 | 11.40 [10.80,12.50] | 11.40 [10.70,12.48] | 0.943 | 11.50 [10.88,12.80] | 11.20 [10.90,11.75] | 0.333 |
| RBCC [x10^12^/L] | 4.12 [3.66,4.56] | 4.01 [3.45,4.31] | 0.006 | 4.12 [3.59,4.54] | 4.14 [3.43,4.52] | 0.769 | 4.07 [3.57,4.62] | 4.11 [3.51,4.80] | 0.684 | 4.06 [3.62,4.56] | 4.18 [3.60,4.77] | 0.935 |
| Sodium [mmol/L] | 137.00 [133.00,140.00] | 136.00 [131.00,139.00] | 0.01 | 137.00 [134.00,140.00] | 137.00 [134.00,141.00] | 0.431 | 138.00 [134.00,141.00] | 137.00 [133.00,141.00] | 0.99 | 139.00 [134.00,143.00] | 139.50 [135.25,144.75] | 0.905 |
| Urea [mmol/L] | 8.10 [5.80,11.80] | 9.10 [6.80,14.20] | 0.015 | 8.70 [6.30,12.90] | 8.75 [6.47,11.93] | 0.798 | 9.60 [6.90,15.50] | 11.35 [7.25,20.42] | 0.083 | 10.85 [7.20,16.78] | 11.65 [7.38,22.40] | 0.484 |
| WCC [x10^9^/L] | 10.18 [7.55,13.92] | 9.43 [7.06,12.32] | 0.081 | 10.55 [8.02,14.41] | 10.25 [8.79,14.83] | 0.611 | 11.46 [8.32,16.22] | 10.80 [7.30,17.42] | 0.531 | 11.67 [8.76,16.07] | 11.28 [8.56,17.07] | 0.709 |
| eGFR [mL/min/1.73m^2^] | 59.00 [39.00,78.00] | 55.00 [35.50,79.00] | 0.594 | 56.50 [34.75,77.00] | 64.00 [42.00,75.50] | 0.438 | 54.00 [33.75,75.00] | 43.00 [26.50,62.50] | 0.028 | 52.00 [34.00,73.00] | 32.00 [30.00,67.00] | 0.175 |
| MCHC[g/L] | 329.00 [319.00,338.00] | 329.00 [319.00,339.00] | 0.873 | 328.00 [317.75,337.00] | 329.00 [317.00,342.25] | 0.555 | 327.00 [316.00,336.00] | 323.50 [313.75,332.00] | 0.081 | 324.00 [310.25,332.00] | 319.50 [313.50,331.00] | 0.709 |
| MCH[pg] | 30.40 [28.90,31.80] | 30.60 [29.20,32.40] | 0.041 | 30.40 [28.90,32.00] | 30.85 [29.10,32.23] | 0.325 | 30.60 [29.10,32.00] | 29.70 [28.35,31.33] | 0.012 | 30.40 [28.70,31.73] | 29.60 [28.20,31.53] | 0.459 |
| MUST score |  |  | <0.001* |  |  | 0.001* |  |  | 0.004 |  |  | 0.651 |
| Low risk | 2245 (69.1) | 86 (71.1) |  | 791 (67.8) | 35 (62.5) |  | 506 (61.2) | 40 (66.7) |  | 109 (54.8) | 12 (66.7) |  |
| Medium risk | 247 (7.6) | 13 (10.7) |  | 124 (10.6) | 11 (19.6) |  | 109 (13.2) | 10 (16.7) |  | 16 (8.0) | 2 (11.1) |  |
| High risk | 214 (6.6) | 20 (16.5) |  | 66 (5.7) | 8 (14.3) |  | 50 (6.0) | 8 (13.3) |  | 18 (9.0) | 1 (5.6) |  |
| Missing | 541 (16.7) | 2 (1.7) |  | 185 (15.9) | 2 (3.6) |  | 162 (19.6) | 2 (3.3) |  | 56 (28.1) | 3 (16.7) |  |
| Mobility score |  |  | <0.001* |  |  | 0.548 |  |  | 0.428 |  |  | 0.037 |
| Fully mobile | 439 (13.5) | 9 (7.4) |  | 33 (2.8) | 1 (1.8) |  | 17 (2.1) | 0 (0.0) |  | 2 (1.0) | 1 (5.6) |  |
| Uses mobility aids | 1658 (51.1) | 61 (50.4) |  | 377 (32.3) | 17 (30.4) |  | 146 (17.7) | 15 (25.0) |  | 4 (2.0) | 2 (11.1) |  |
| Partial weight bearing | 337 (10.4) | 15 (12.4) |  | 187 (16.0) | 7 (12.5) |  | 89 (10.8) | 7 (11.7) |  | 11 (5.5) | 0 (0.0) |  |
| Non-weight bearing | 179 (5.5) | 16 (13.2) |  | 180 (15.4) | 12 (21.4) |  | 121 (14.6) | 10 (16.7) |  | 13 (6.5) | 2 (11.1) |  |
| Immobile | 219 (6.7) | 13 (10.7) |  | 244 (20.9) | 15 (26.8) |  | 347 (42.0) | 24 (40.0) |  | 118 (59.3) | 12 (66.7) |  |
| Missing | 415 (12.8) | 7 (5.8) |  | 145 (12.4) | 4 (7.1) |  | 107 (12.9) | 4 (6.7) |  | 51 (25.6) | 1 (5.6) |  |
| Length of stay | 7.00 [3.00,14.00] | 17.00 [11.00,31.00] | <0.001* | 9.00 [4.00,17.00] | 19.50 [13.75,31.75] | <0.001* | 9.00 [4.00,16.00] | 20.00 [12.75,29.75] | <0.001* | 6.00 [3.00,11.50] | 13.50 [9.25,16.50] | <0.001* |

Based on data for 10,504 patients, excluding columns for ‘no risk’ and for missing Braden score. Reporting n(%) or median (IQR). Row percentages. * Indicating p≤0.002. . ALP, alkaline phosphatase; BMI, body mass index; APTT, activated partial thromboplastin time; CRP, C-reactive protein; eGFR, estimated glomerular filtration rate; MCH, mean cell haemoglobin; MCHC, mean cell haemoglobin concentration INR, International normalised ratio; MCV, mean cell volume; MPV, mean platelet volume; RBCC, red blood cell count; WCC, white cell count; MUST, Malnutrition Universal Screening Tool. Mobility codes: 0 fully mobile, 1 mobility assistance (frame/walking aid), 2 partially weight bearing; 3 non-weight bearing, sit with support, 4 Immobile/bedridden.

Normal ranges: APTT 20-30; Albumin 32-50; ALP 30-130; CRP 0-5; Creatinine 49-90 (females), 64-104 (males); Haematocrit 0.36-0.46 (females), 0.40-0.50 (males); Haemoglobin 120-165 (females), 130-170 (males); INR 0.8-1.2; MCV 83-101; MPV 9-13; Platelets 150-400; Prothrombin time 9-12; RBCC 3.8-5.0 (females), 4.5-6.2 (males); Sodium 135-145; Urea 2.5-7.8 WCC 3.7-11.0; eGFR >60; MCHC 315-360; MCH 27.0-32.0;

Supplementary Table 3. Characteristics of patient admissions, stratified by Malnutrition Universal Screening Tool category and hospital-acquired pressure injury incidence

|  | Low risk |  | p | Medium risk |  | p | High risk |  | p |
| --- | --- | --- | --- | --- | --- | --- | --- | --- | --- |
|  | No HAPI | HAPI |  | No HAPI | HAPI |  | No HAPI | HAPI |  |
| N* | 6453 | 197 |  | 683 | 41 |  | 538 | 43 |  |
| Age | 78.00 [65.00,86.00] | 84.00 [77.00,89.00] | <0.001* | 83.00 [73.00,89.00] | 86.00 [81.00,93.00] | 0.004 | 78.00 [66.00,86.00] | 84.00 [76.00,86.00] | 0.009 |
| Age group |  |  | <0.001* |  |  | 0.085 |  |  | 0.010 |
| < 41 years | 365 (5.7) | 0 (0.0) |  | 28 (4.1) | 0 (0.0) |  | 42 (7.8) | 0 (0.0) |  |
| 41 to 60 years | 830 (12.9) | 9 (4.6) |  | 51 (7.5) | 2 (4.9) |  | 61 (11.3) | 0 (0.0) |  |
| 61 to 80 years | 2559 (39.7) | 68 (34.5) |  | 221 (32.4) | 8 (19.5) |  | 198 (36.8) | 16 (37.2) |  |
| > 80 years | 2699 (41.8) | 120 (60.9) |  | 383 (56.1) | 31 (75.6) |  | 237 (44.1) | 27 (62.8) |  |
| Gender |  |  | 0.088 |  |  |  |  |  | 0.843 |
| Female | 3123 (48.4) | 108 (54.8) |  | 373 (54.6) | 29 (70.7) | 0.064 | 348 (64.7) | 29 (67.4) |  |
| Male | 3330 (51.6) | 89 (45.2) |  | 310 (45.4) | 12 (29.3) | 0.064 | 190 (35.3) | 14 (32.6) |  |
| Ethnic group |  |  | 0.059 |  |  | 0.196 |  |  | 0.271 |
| White British | 4886 (75.7) | 159 (80.7) |  | 515 (75.4) | 35 (85.4) |  | 404 (75.1) | 37 (86.0) |  |
| Not White British | 525 (8.1) | 7 (3.6) |  | 40 (5.9) | 0 (0.0) |  | 46 (8.6) | 2 (4.7) |  |
| Missing | 1042 (16.1) | 31 (15.7) |  | 128 (18.7) | 6 (14.6) |  | 88 (16.4) | 4 (9.3) |  |
| BMI category |  |  | <0.001* |  |  | 0.003 |  |  | 0.722 |
| Underweight | 26 (0.4) | 5 (2.5) |  | 70 (10.2) | 12 (29.3) |  | 437 (81.2) | 38 (88.4) |  |
| Healthy weight | 2294 (35.5) | 87 (44.2) |  | 492 (72.0) | 23 (56.1) |  | 73 (13.6) | 4 (9.3) |  |
| Overweight | 2071 (32.1) | 55 (27.9) |  | 53 (7.8) | 1 (2.4) |  | 14 (2.6) | 0 (0.0) |  |
| Obese | 1879 (29.1) | 44 (22.3) |  | 25 (3.7) | 1 (2.4) |  | 12 (2.2) | 1 (2.3) |  |
| Missing | 183 (2.8) | 6 (3.0) |  | 43 (6.3) | 4 (9.8) |  | 2 (0.4) | 0 (0.0) |  |
| APTT [secs] | 25.50 [23.30,28.50] | 26.50 [23.67,29.95] | 0.006 | 25.65 [23.40,28.90] | 25.85 [23.10,27.95] | 0.676 | 26.10 [23.90,28.75] | 26.50 [23.75,28.37] | 0.694 |
| Albumin [g/L] | 35.00 [30.00,38.00] | 32.00 [27.75,36.00] | <0.001* | 33.00 [28.00,37.00] | 32.00 [27.00,34.00] | 0.133 | 33.00 [28.00,37.00] | 30.00 [26.00,35.00] | 0.025 |
| ALP [IU/L] | 101.00 [79.00,135.00] | 111.50 [87.75,157.00] | 0.002* | 107.00 [84.00,145.00] | 93.00 [74.00,133.00] | 0.059 | 104.00 [82.00,137.00] | 96.00 [79.50,139.50] | 0.404 |
| CRP [mg/L] | 32.20 [6.80,109.40] | 54.80 [16.98,123.37] | <0.001* | 45.95 [9.30,120.88] | 30.50 [10.80,66.30] | 0.345 | 33.80 [7.32,110.38] | 29.40 [7.30,127.80] | 0.899 |
| CRP to Albumin [mg/g] | 0.95 [0.19,3.67] | 1.68 [0.50,4.17] | <0.001* | 1.45 [0.25,4.15] | 0.87 [0.33,3.41] | 0.455 | 1.08 [0.21,3.67] | 0.94 [0.21,4.38] | 0.989 |
| Creatinine [µmol/L] | 84.00 [64.00,117.00] | 96.00 [69.00,142.25] | 0.004 | 80.00 [61.00,115.00] | 83.00 [60.00,127.00] | 0.725 | 70.00 [53.00,96.00] | 68.00 [51.00,91.00] | 0.58 |
| Haematocrit [L/L] | 0.39 [0.35,0.42] | 0.37 [0.34,0.41] | 0.02 | 0.37 [0.34,0.41] | 0.37 [0.31,0.41] | 0.465 | 0.38 [0.34,0.42] | 0.38 [0.32,0.41] | 0.435 |
| Haemoglobin [g/L] | 128.00 [113.00,141.00] | 124.00 [108.00,136.00] | 0.006 | 123.50 [111.00,137.00] | 124.00 [103.00,135.00] | 0.491 | 124.00 [109.00,137.00] | 122.00 [105.00,131.50] | 0.267 |
| INR [-] | 1.10 [1.00,1.10] | 1.10 [1.00,1.20] | <0.001* | 1.10 [1.00,1.20] | 1.10 [1.00,1.20] | 0.853 | 1.10 [1.00,1.10] | 1.10 [1.00,1.20] | 0.805 |
| MCV [fL] | 91.80 [87.70,96.10] | 92.30 [88.00,96.70] | 0.165 | 92.55 [88.50,97.20] | 93.30 [89.40,97.90] | 0.382 | 92.50 [88.10,97.50] | 94.70 [91.00,100.65] | 0.071 |
| MPV [fL] | 10.10 [9.50,10.90] | 10.20 [9.40,11.00] | 0.976 | 10.10 [9.50,10.80] | 10.30 [9.60,11.10] | 0.312 | 10.00 [9.30,10.80] | 10.10 [9.55,10.70] | 0.632 |
| Platelets [x10^9^/L] | 243.00 [188.00,310.00] | 233.00 [181.00,312.50] | 0.77 | 252.00 [186.00,334.00] | 251.00 [183.00,334.00] | 0.928 | 258.00 [206.00,341.00] | 243.00 [160.50,308.00] | 0.242 |
| Prothrombin time [secs] | 11.10 [10.50,11.90] | 11.40 [10.80,12.40] | <0.001* | 11.20 [10.60,12.00] | 11.35 [10.40,12.45] | 0.961 | 11.10 [10.60,11.90] | 11.00 [10.53,12.15] | 0.52 |
| RBCC [x10^12^/L] | 4.22 [3.73,4.68] | 4.08 [3.55,4.59] | 0.017 | 4.03 [3.60,4.52] | 3.82 [3.32,4.63] | 0.417 | 4.09 [3.59,4.54] | 3.97 [3.43,4.31] | 0.125 |
| Sodium [mmol/L] | 137.00 [134.00,140.00] | 136.00 [132.00,139.00] | 0.132 | 137.00 [133.00,140.00] | 137.00 [134.00,140.00] | 0.546 | 136.00 [133.00,139.00] | 135.00 [132.00,138.00] | 0.069 |
| Urea [mmol/L] | 7.60 [5.40,11.20] | 9.30 [7.00,15.70] | <0.001* | 8.20 [5.50,12.60] | 10.00 [6.50,15.10] | 0.146 | 7.60 [5.30,11.40] | 7.80 [5.80,10.95] | 0.674 |
| WCC [x10^9^/L] | 10.16 [7.63,13.80] | 10.16 [7.91,14.13] | 0.558 | 10.65 [7.62,14.86] | 9.08 [7.75,16.56] | 0.794 | 9.88 [6.98,14.45] | 10.03 [7.22,14.50] | 0.967 |
| eGFR [mL/min/1.73m^2^] | 60.00 [40.00,79.00] | 51.50 [32.00,70.75] | 0.001* | 59.00 [39.00,78.00] | 60.00 [31.25,78.75] | 0.867 | 67.00 [46.00,82.00] | 71.50 [48.00,82.00] | 0.737 |
| MCHC[g/L] | 330.00 [320.00,339.00] | 327.00 [317.00,338.00] | 0.011 | 330.00 [319.00,338.25] | 330.00 [318.00,336.00] | 0.899 | 328.00 [319.00,339.00] | 329.00 [318.00,337.50] | 0.806 |
| MCH [pg] | 30.40 [28.90,31.80] | 30.30 [28.90,31.80] | 0.771 | 30.50 [29.00,32.10] | 30.60 [29.50,32.00] | 0.561 | 30.60 [28.90,32.10] | 31.10 [29.90,32.70] | 0.134 |
| Braden score |  |  | <0.001* |  |  | 0.165 |  |  | 0.057 |
| No risk | 2754 (42.7) | 24 (12.2) |  | 184 (26.9) | 5 (12.2) |  | 186 (34.6) | 6 (14.0) |  |
| Low risk | 2245 (34.8) | 86 (43.7) |  | 247 (36.2) | 13 (31.7) |  | 214 (39.8) | 20 (46.5) |  |
| Moderate risk | 791 (12.3) | 35 (17.8) |  | 124 (18.2) | 11 (26.8) |  | 66 (12.3) | 8 (18.6) |  |
| High risk | 506 (7.8) | 40 (20.3) |  | 109 (16.0) | 10 (24.4) |  | 50 (9.3) | 8 (18.6) |  |
| Severe risk | 109 (1.7) | 12 (6.1) |  | 16 (2.3) | 2 (4.9) |  | 18 (3.3) | 1 (2.3) |  |
| Missing | 48 (0.7) | 0 (0.0) |  | 3 (0.4) | 0 (0.0) |  | 4 (0.7) | 0 (0.0) |  |
| Mobility score |  |  | <0.001* |  |  | 0.312 |  |  | 0.054 |
| Fully mobile | 2011 (31.2) | 10 (5.1) |  | 141 (20.6) | 4 (9.8) |  | 136 (25.3) | 3 (7.0) |  |
| Uses mobility aids | 2311 (35.8) | 77 (39.1) |  | 250 (36.6) | 16 (39.0) |  | 220 (40.9) | 23 (53.5) |  |
| Partial weight bearing | 526 (8.2) | 19 (9.6) |  | 67 (9.8) | 4 (9.8) |  | 42 (7.8) | 7 (16.3) |  |
| Non-weight bearing | 383 (5.9) | 35 (17.8) |  | 57 (8.3) | 5 (12.2) |  | 38 (7.1) | 2 (4.7) |  |
| Immobile | 671 (10.4) | 45 (22.8) |  | 104 (15.2) | 10 (24.4) |  | 60 (11.2) | 5 (11.6) |  |
| Missing | 551 (8.5) | 11 (5.6) |  | 64 (9.4) | 2 (4.9) |  | 42 (7.8) | 3 (7.0) |  |
| Length of stay | 6.00 [3.00,13.00] | 18.00 [11.00,31.00] | <0.001* | 8.00 [4.00,16.00] | 18.00 [13.00,31.00] | <0.001* | 8.00 [4.00,15.00] | 20.00 [13.50,27.50] | <0.001* |

Based on data for 10,504 patients, excluding columns for missing MUST score. Reporting n(%) or median (IQR). Row percentages.* Indicating p≤0.002. . ALP, alkaline phosphatase; BMI, body mass index; APTT, activated partial thromboplastin time; CRP, C-reactive protein; eGFR, estimated glomerular filtration rate; MCH, mean cell haemoglobin; MCHC, mean cell haemoglobin concentration INR, International normalised ratio; MCV, mean cell volume; MPV, mean platelet volume; RBCC, red blood cell count; WCC, white cell count; MUST, Malnutrition Universal Screening Tool. Mobility codes: 0 fully mobile, 1 mobility assistance (frame/walking aid), 2 partially weight bearing; 3 non-weight bearing, sit with support, 4 Immobile/bedridden.

Normal ranges: APTT 20-30; Albumin 32-50; ALP 30-130; CRP 0-5; Creatinine 49-90 (females), 64-104 (males); Haematocrit 0.36-0.46 (females), 0.40-0.50 (males); Haemoglobin 120-165 (females), 130-170 (males); INR 0.8-1.2; MCV 83-101; MPV 9-13; Platelets 150-400; Prothrombin time 9-12; RBCC 3.8-5.0 (females), 4.5-6.2 (males); Sodium 135-145; Urea 2.5-7.8 WCC 3.7-11.0; eGFR >60; MCHC 315-360; MCH 27.0-32.0;

Supplementary Table 4. Characteristics of patient admissions, stratified by Mobility score category and hospital-acquired pressure injury incidence

|  | Fully mobile |  | p | Uses mobility aids |  | p | Partial weight bearing |  | p | Non-weight bearing |  | p | Immobile |  | p |
| --- | --- | --- | --- | --- | --- | --- | --- | --- | --- | --- | --- | --- | --- | --- | --- |
|  | No HAPI | HAPI |  | No HAPI | HAPI |  | No HAPI | HAPI |  | No HAPI | HAPI |  | No HAPI | HAPI |  |
| N* | 2777 | 18 |  | 3185 | 119 |  | 704 | 30 |  | 530 | 43 |  | 992 | 65 |  |
| Age | 66.00 [51.00, 77.00] | 85.50 [74.50, 88.00] | <0.001* | 82.00 [74.00, 88.00] | 84.00 [79.00, 90.00] | 0.001* | 82.00 [74.00, 88.00] | 85.00 [80.25, 87.00] | 0.201 | 84.00 [75.00, 89.00] | 85.00 [77.00, 88.50] | 0.945 | 82.00 [74.00, 88.00] | 82.00 [74.00, 90.00] | 0.576 |
| Age group |  |  | <0.001* |  |  | 0.004 |  |  | 0.221 |  |  | 0.783 |  |  | 0.682 |
| < 41 years | 397 (14.3) | 0 (0.0) |  | 50 (1.6) | 0 (0.0) |  | 15 (2.1) | 0 (0.0) |  | 9 (1.7) | 0 (0.0) |  | 16 (1.6) | 0 (0.0) |  |
| 41 to 60 years | 710 (25.6) | 1 (5.6) |  | 217 (6.8) | 0 (0.0) |  | 43 (6.1) | 2 (6.7) |  | 26 (4.9) | 3 (7.0) |  | 63 (6.4) | 5 (7.7) |  |
| 61 to 80 years | 1211 (43.6) | 6 (33.3) |  | 1162 (36.5) | 39 (32.8) |  | 255 (36.2) | 6 (20.0) |  | 177 (33.4) | 14 (32.6) |  | 364 (36.7) | 26 (40.0) |  |
| >80 years | 459 (16.5) | 11 (61.1) |  | 1756 (55.1) | 80 (67.2) |  | 391 (55.5) | 22 (73.3) |  | 318 (60.0) | 26 (60.5) |  | 549 (55.3) | 34 (52.3) |  |
| Gender |  |  | 1 |  |  | 0.206 |  |  | 0.918 |  |  | 0.258 |  |  | 0.106 |
| Female | 1304 (47.0) | 8 (44.4) |  | 1726 (54.2) | 72 (60.5) |  | 333 (47.3) | 15 (50.0) |  | 254 (47.9) | 25 (58.1) |  | 515 (51.9) | 41 (63.1) |  |
| Male | 1473 (53.0) | 10 (55.6) |  | 1459 (45.8) | 47 (39.5) |  | 371 (52.7) | 15 (50.0) |  | 276 52.1) | 18 (41.9) |  | 477 (48.1) | 24 (36.9) |  |
| Ethnic group |  |  | 0.064 |  |  | 0.324 |  |  | 0.672 |  |  | 0.204 |  |  | 0.828 |
| White British | 1919 (69.1) | 17 (94.4) |  | 2517 (79.0) | 100 (84.0) |  | 557 (79.1) | 23 (76.7) |  | 425 (80.2) | 38 (88.4) |  | 758 (76.4) | 48 (73.8) |  |
| Not White British | 332 (12.0) | 0 (0.0) |  | 198 (6.2) | 4 (3.4) |  | 41 (5.8) | 1 (3.3) |  | 34 (6.4) | 0 (0.0) |  | 65 (6.6) | 4 (6.2) |  |
| Missing | 526 (18.9) | 1 (5.6) |  | 470 (14.8) | 15 (12.6) |  | 106 (15.1) | 6 (20.0) |  | 71 (13.4) | 5 (11.6) |  | 169 (17.0) | 13 (20.0) |  |
| BMI category |  |  | 0.006 |  |  | <0.001* |  |  | <0.001* |  |  | 0.186 |  |  | 0.258 |
| Underweight | 115 (4.1) | 3 (16.7) |  | 231 (7.3) | 25 (21.0) |  | 53 (7.5) | 10 (33.3) |  | 48 (9.1) | 4 (9.3) |  | 68 (6.9) | 9 (13.8) |  |
| Healthy weight | 863 (31.1) | 10 (55.6) |  | 1172 (36.8) | 45 (37.8) |  | 273 (38.8) | 13 (43.3) |  | 186 (35.1) | 19 (44.2) |  | 386 (38.9) | 21 (32.3) |  |
| Overweight | 802 (28.9) | 3 (16.7) |  | 853 (26.8) | 28 (23.5) |  | 152 (21.6) | 5 (16.7) |  | 135 (25.5) | 6 (14.0) |  | 216 (21.8) | 16 (24.6) |  |
| Obese | 744 (26.8) | 2 (11.1) |  | 706 (22.2) | 17 (14.3) |  | 157 (22.3) | 2 (6.7) |  | 116 (21.9) | 13 (30.2) |  | 174 (17.5) | 11 (16.9) |  |
| Missing | 253 (9.1) | 0 (0.0) |  | 223 (7.0) | 4 (3.4) |  | 69 (9.8) | 0 (0.0) |  | 45 (8.5) | 1 (2.3) |  | 148 (14.9) | 8 (12.3) |  |
| APTT [secs] | 25.00 [22.90, 27.40] | 26.90 [24.20, 32.80] | 0.035 | 25.80 [23.40, 28.80] | 26.65 [23.65, 29.68] | 0.182 | 26.05 [23.63, 29.28] | 27.90 [25.75, 30.70] | 0.06 | 26.10 [23.60, 29.78] | 25.80 [22.65, 28.95] | 0.403 | 26.30 [23.45, 29.70] | 25.90 [23.60, 28.90] | 0.972 |
| Albumin [g/L] | 37.00 [32.00, 41.00] | 31.00 [25.75, 37.75] | 0.004 | 34.00 [30.00, 37.00] | 33.00 [28.00, 36.00] | 0.042 | 34.00 [29.00, 38.00] | 30.00 [27.25, 33.00] | 0.012 | 32.00 [28.00, 36.00] | 32.00 [28.50, 34.00] | 0.476 | 31.00 [27.00, 36.00] | 30.00 [26.00, 34.00] | 0.053 |
| ALP [IU/L] | 94.00 [76.00, 125.00] | 113.00 [91.25, 174.75] | 0.029 | 104.00 [82.00, 138.00] | 102.50 [80.00, 136.50] | 0.575 | 105.00 [81.00, 142.25] | 108.50 [93.75, 125.50] | 0.79 | 108.50 [85.00, 153.00] | 131.00 [92.50, 159.50] | 0.201 | 109.00 [84.50, 146.00] | 108.00 [79.00, 157.00] | 0.803 |
| CRP [mg/L] | 22.30 [4.20, 95.62] | 15.05 [2.15, 155.40] | 0.989 | 34.70 [8.00, 109.15] | 40.50 [14.75, 98.00] | 0.493 | 34.85 [8.72, 111.22] | 26.70 [10.90, 103.08] | 0.994 | 48.20 [9.90, 126.28] | 66.30 [13.90, 139.10] | 0.527 | 56.60 [15.60, 143.30] | 67.45 [22.30, 183.62] | 0.137 |
| CRP to Albumin [mg/g] | 0.63 [0.11, 2.90] | 0.48 [0.07, 5.23] | 0.838 | 1.05 [0.23, 3.67] | 1.30 [0.43, 3.13] | 0.392 | 1.05 [0.26, 3.75] | 0.91 [0.37, 3.69] | 0.926 | 1.59 [0.28, 4.57] | 1.95 [0.44, 4.80] | 0.564 | 1.86 [0.48, 5.20] | 2.38 [0.63, 7.75] | 0.11 |
| Creatinine [µmol/L] | 76.00 [61.00, 98.00] | 91.00 [74.25, 145.50] | 0.108 | 85.00 [64.00, 121.00] | 80.00 [62.00, 106.50] | 0.113 | 87.00 [63.00, 122.00] | 86.50 [60.50, 154.25] | 0.86 | 87.00 [64.00, 129.00] | 91.00 [71.50, 134.00] | 0.29 | 86.00 [63.00, 130.75] | 94.00 [67.75, 153.75] | 0.303 |
| Haematocrit [L/L] | 0.40 [0.36, 0.44] | 0.38 [0.32, 0.42] | 0.056 | 0.38 [0.34, 0.41] | 0.37 [0.33, 0.40] | 0.394 | 0.38 [0.34, 0.42] | 0.38 [0.34, 0.40] | 0.928 | 0.38 [0.34, 0.42] | 0.40 [0.34, 0.43] | 0.491 | 0.38 [0.34, 0.42] | 0.36 [0.31, 0.41] | 0.119 |
| Haemoglobin [g/L] | 133.00 [119.00, 146.00] | 119.50 [103.75, 136.50] | 0.031 | 124.00 [110.00, 137.00] | 125.00 [108.50, 133.00] | 0.364 | 125.00 [111.00, 138.00] | 125.50 [107.00, 134.50] | 0.646 | 126.00 [111.00, 137.00] | 128.00 [110.00, 139.50] | 0.471 | 123.00 [109.00, 138.00] | 116.00 [99.00, 137.00] | 0.127 |
| INR [-] | 1.00 [1.00, 1.10] | 1.20 [1.10, 1.30] | 0.001* | 1.10 [1.00, 1.10] | 1.10 [1.00, 1.20] | 0.281 | 1.10 [1.00, 1.20] | 1.10 [1.10, 1.20] | 0.017 | 1.10 [1.00, 1.20] | 1.10 [1.00, 1.20] | 0.821 | 1.10 [1.00, 1.20] | 1.10 [1.00, 1.10] | 0.228 |
| MCV [fL] | 90.70 [86.80, 94.70] | 96.70 [92.87, 100.07] | <0.001* | 92.20 [88.20, 96.50] | 91.60 [88.15, 96.85] | 0.781 | 92.60 [88.70, 96.90] | 92.90 [89.40, 96.52] | 0.664 | 92.40 [88.20, 96.57] | 93.20 [89.30, 98.10] | 0.393 | 93.20 [88.70, 97.70] | 93.40 [87.90, 99.20] | 0.556 |
| MPV [fL] | 10.10 [9.50, 10.80] | 10.70 [9.83, 11.33] | 0.076 | 10.20 [9.50, 10.90] | 10.20 [9.40, 10.70] | 0.147 | 10.10 [9.50, 10.90] | 10.10 [9.33, 10.78] | 0.489 | 10.10 [9.40, 10.80] | 10.10 [9.55, 10.80] | 0.629 | 10.30 [9.60, 11.10] | 10.25 [9.40, 11.22] | 0.655 |
| Platelets [x10^9^/L] | 247.00 [193.00, 317.00] | 213.50 [138.00, 241.00] | 0.022 | 245.00 [190.00, 315.25] | 233.00 [183.50, 307.00] | 0.521 | 237.00 [188.00, 309.75] | 263.50 [217.75, 336.25] | 0.157 | 254.00 [194.00, 314.00] | 251.00 [180.00, 346.50] | 0.548 | 245.00 [184.00, 325.00] | 224.00 [169.00, 354.00] | 0.597 |
| Prothrombin time [secs] | 10.90 [10.40, 11.60] | 12.40 [11.20, 13.00] | 0.002* | 11.10 [10.60, 11.90] | 11.40 [10.60, 12.50] | 0.185 | 11.30 [10.70, 12.10] | 11.50 [11.40, 12.40] | 0.031 | 11.40 [10.80, 12.40] | 11.40 [10.80, 12.55] | 0.792 | 11.40 [10.70, 12.40] | 11.40 [10.60, 11.90] | 0.355 |
| RBCC [x10^12^/L] | 4.42 [3.92, 4.84] | 3.93 [3.26, 4.31] | 0.003 | 4.08 [3.62, 4.53] | 4.05 [3.54, 4.42] | 0.402 | 4.10 [3.64, 4.56] | 4.21 [3.80, 4.50] | 0.904 | 4.14 [3.66, 4.53] | 4.20 [3.54, 4.84] | 0.521 | 4.09 [3.61, 4.60] | 3.84 [3.31, 4.63] | 0.09 |
| Sodium [mmol/L] | 137.00 [134.00, 139.00] | 138.00 [134.25, 140.75] | 0.588 | 137.00 [133.00, 140.00] | 135.00 [131.00, 138.00] | 0.001* | 137.00 [134.00, 140.00] | 135.50 [132.00, 139.00] | 0.221 | 137.00 [134.00, 140.00] | 137.00 [135.50, 139.00] | 0.581 | 138.00 [134.00, 141.00] | 138.00 [134.00, 141.00] | 0.637 |
| Urea [mmol/L] | 6.10 [4.50, 8.50] | 8.30 [5.32, 11.23] | 0.066 | 8.20 [5.80, 11.70] | 8.50 [6.40, 11.30] | 0.455 | 8.20 [6.00, 11.88] | 10.20 [6.90, 16.00] | 0.083 | 9.00 [6.20, 13.30] | 9.40 [7.25, 17.80] | 0.091 | 9.40 [6.50, 14.40] | 11.40 [7.30, 20.70] | 0.051 |
| WCC [x10^9^/L] | 9.89 [7.39, 13.23] | 9.52 [8.17, 12.90] | 0.601 | 10.05 [7.50, 13.77] | 9.88 [7.50, 14.21] | 0.921 | 10.48 [7.84, 14.40] | 9.53 [7.86, 12.16] | 0.586 | 11.07 [8.30, 15.58] | 10.70 [8.48, 13.93] | 0.68 | 11.16 [8.27, 15.29] | 11.42 [7.89, 17.21] | 0.853 |
| eGFR [mL/min/1.73m2] | 68.00 [50.00, 82.00] | 49.50 [32.00, 76.00] | 0.094 | 58.00 [39.00, 77.75] | 62.00 [44.00, 81.00] | 0.156 | 58.00 [37.00, 78.00] | 51.00 [31.50, 77.50] | 0.492 | 57.50 [36.00, 77.00] | 51.50 [34.00, 68.75] | 0.332 | 56.00 [34.00, 77.00] | 46.50 [28.25, 67.75] | 0.031 |
| MCHC [g/L] | 334.00 [325.00, 343.00] | 330.00 [318.50, 339.75] | 0.17 | 329.00 [319.00, 338.00] | 329.00 [319.00, 338.00] | 0.922 | 329.00 [318.00, 338.00] | 327.00 [317.50, 330.75] | 0.296 | 329.00 [318.00, 337.75] | 327.00 [318.00, 338.00] | 0.936 | 325.00 [314.00, 335.00] | 325.00 [313.00, 333.00] | 0.639 |
| MCH [pg] | 30.40 [28.90, 31.80] | 31.70 [30.25, 33.15] | 0.013 | 30.40 [28.90, 32.00] | 30.30 [29.20, 31.55] | 0.677 | 30.50 [29.00, 31.90] | 30.45 [29.25, 31.80] | 0.905 | 30.30 [28.90, 31.70] | 30.60 [28.70, 32.25] | 0.42 | 30.40 [28.80, 31.80] | 30.20 [28.50, 32.30] | 0.881 |
| Braden score |  |  | <0.001* |  |  | <0.001* |  |  | 0.439 |  |  | 0.893 |  |  | 0.436 |
| No risk | 2254 (81.2) | 7 (38.9) |  | 964 (30.3) | 24 (20.2) |  | 78 (11.1) | 1 (3.3) |  | 33 (6.2) | 3 (7.0) |  | 52 (5.2) | 1 (1.5) |  |
| Low risk | 439 (15.8) | 9 (50.0) |  | 1658 (52.1) | 61 (51.3) |  | 337 (47.9) | 15 (50.0) |  | 179 (33.8) | 16 (37.2) |  | 219 (22.1) | 13 (20.0) |  |
| Moderate risk | 33 (1.2) | 1 (5.6) |  | 377 (11.8) | 17 (14.3) |  | 187 (26.6) | 7 (23.3) |  | 180 (34.0) | 12 (27.9) |  | 244 (24.6) | 15 (23.1) |  |
| High risk | 17 (0.6) | 0 (0.0) |  | 146 (4.6) | 15 (12.6) |  | 89 (12.6) | 7 (23.3) |  | 121 (22.8) | 10 (23.3) |  | 347 (35.0) | 24 (36.9) |  |
| Severe risk | 2 (0.1) | 1 (5.6) |  | 4 (0.1) | 2 (1.7) |  | 11 (1.6) | 0 (0.0) |  | 13 (2.5) | 2 (4.7) |  | 118 (11.9) | 12 (18.5) |  |
| Missing | 32 (1.2) | 0 (0.0) |  | 36 (1.1) | 0 (0.0) |  | 2 (0.3) | 0 (0.0) |  | 4 (0.8) | 0 (0.0) |  | 12 (1.2) | 0 (0.0) |  |
| MUST score |  |  | 0.001* |  |  | <0.001* |  |  | 0.001* |  |  | 0.354 |  |  | 0.232 |
| Low risk | 2011 (72.4) | 10 (55.6) |  | 2311 (72.6) | 77 (64.7) |  | 526 (74.7) | 19 (63.3) |  | 383 (72.3) | 35 (81.4) |  | 671 (67.6) | 45 (69.2) |  |
| Medium risk | 141 (5.1) | 4 (22.2) |  | 250 (7.8) | 16 (13.4) |  | 67 (9.5) | 4 (13.3) |  | 57 (10.8) | 5 (11.6) |  | 104 (10.5) | 10 (15.4) |  |
| High risk | 136 (4.9) | 3 (16.7) |  | 220 (6.9) | 23 (19.3) |  | 42 (6.0) | 7 (23.3) |  | 38 (7.2) | 2 (4.7) |  | 60 (6.0) | 5 (7.7) |  |
| Missing | 489 (17.6) | 1 (5.6) |  | 404 (12.7) | 3 (2.5) |  | 69 (9.8) | 0 (0.0) |  | 52 (9.8) | 1 (2.3) |  | 157 (15.8) | 5 (7.7) |  |
| Length of stay | 4.00 [2.00, 7.00] | 16.50 [11.00, 32.50] | <0.001* | 7.00 [4.00, 14.00] | 19.00 [13.00, 29.00] | <0.001* | 10.00 [5.00, 17.25] | 18.50 [11.25, 42.75] | <0.001* | 10.00 [5.00, 18.00] | 20.00 [14.50, 31.00] | <0.001* | 9.00 [5.00, 17.00] | 18.00 [11.00, 25.00] | <0.001* |

Based on data for 10,504 patients, excluding columns for missing mobility score. Reporting n(%) or median (IQR). Row percentages. * Indicating p≤0.002. . ALP, alkaline phosphatase; BMI, body mass index; APTT, activated partial thromboplastin time; CRP, C-reactive protein; eGFR, estimated glomerular filtration rate; MCH, mean cell haemoglobin; MCHC, mean cell haemoglobin concentration INR, International normalised ratio; MCV, mean cell volume; MPV, mean platelet volume; RBCC, red blood cell count; WCC, white cell count; MUST, Malnutrition Universal Screening Tool. Mobility codes: 0 fully mobile, 1 mobility assistance (frame/walking aid), 2 partially weight bearing; 3 non-weight bearing, sit with support, 4 Immobile/bedridden.

Normal ranges: APTT 20-30; Albumin 32-50; ALP 30-130; CRP 0-5; Creatinine 49-90 (females), 64-104 (males); Haematocrit 0.36-0.46 (females), 0.40-0.50 (males); Haemoglobin 120-165 (females), 130-170 (males); INR 0.8-1.2; MCV 83-101; MPV 9-13; Platelets 150-400; Prothrombin time 9-12; RBCC 3.8-5.0 (females), 4.5-6.2 (males); Sodium 135-145; Urea 2.5-7.8 WCC 3.7-11.0; eGFR >60; MCHC 315-360; MCH 27.0-32.0;
